# Dendritic, delayed, stochastic CaMKII activation in behavioural time scale plasticity

**DOI:** 10.1038/s41586-024-08021-8

**Published:** 2024-10-09

**Authors:** Anant Jain, Yoshihisa Nakahata, Tristano Pancani, Tetsuya Watabe, Polina Rusina, Kelly South, Kengo Adachi, Long Yan, Noriko Simorowski, Hiro Furukawa, Ryohei Yasuda

**Affiliations:** 1 Neuronal Signal Transduction Group, Max Planck Florida Institute for Neuroscience, Jupiter, FL USA; 2 W.M. Keck Structural Biology Laboratory, Cold Spring Harbor Laboratory, Cold Spring Harbor, NY USA; 3 Present Address: Centre for High Impact Neuroscience and Translational Applications (CHINTA), TCG CREST, Kolkata, India

**Keywords:** Cellular neuroscience, Long-term potentiation

## Abstract

Behavioural time scale plasticity (BTSP) is non-Hebbian plasticity induced by integrating presynaptic and postsynaptic components separated by a behaviourally relevant time scale (seconds)^1^. BTSP in hippocampal CA1 neurons underlies place cell formation. However, the molecular mechanisms that enable synapse-specific plasticity on a behavioural time scale are unknown. Here we show that BTSP can be induced in a single dendritic spine using two-photon glutamate uncaging paired with postsynaptic current injection temporally separated by a behavioural time scale. Using an improved Ca^2+^/calmodulin-dependent kinase II (CaMKII) sensor, we did not detect CaMKII activation during this BTSP induction. Instead, we observed dendritic, delayed and stochastic CaMKII activation (DDSC) associated with Ca^2+^ influx and plateau potentials 10–100 s after BTSP induction. DDSC required both presynaptic and postsynaptic activity, which suggests that CaMKII can integrate these two signals. Also, optogenetically blocking CaMKII 15–30 s after the BTSP protocol inhibited synaptic potentiation, which indicated that DDSC is an essential mechanism of BTSP. IP_3_-dependent intracellular Ca^2+^ release facilitated both DDSC and BTSP. Thus, our study suggests that non-synapse-specific CaMKII activation provides an instructive signal with an extensive time window over tens of seconds during BTSP.

## Abstract (web summary)

Induction of behavioural time scale plasticity leads to dendritic, delayed and stochastic Ca^2+^/calmodulin-dependent kinase II activation, findings that clarify the mechanisms that underlie place cell formation.

## Main

Synaptic plasticity is the basis of acquiring and storing new information in the brain^2^ and can be induced by specific patterns of electrical activity^3^. In the classical Hebbian mechanism, coordinated presynaptic and postsynaptic activation leads to removal of the Mg^2+^ block in NMDARs. This process allows Ca^2+^ ions to flow into dendritic spines to activate enzymes, including CaMKII, to induce synaptic potentiation^4^. However, computational studies suggest that the Hebbian plasticity rule, which requires temporal coincidence within milliseconds, does not fit with simple learning behaviours that occur over seconds to minutes^5^.

Recently, BTSP was discovered at CA3–CA1 synapses as a mechanism of place cell formation^1,6^. In this type of plasticity, brief postsynaptic depolarization is paired with presynaptic inputs within a behavioural time scale or within hundreds of milliseconds to seconds to induce synaptic potentiation during place cell induction. Synaptic potentiation can be induced either by forward (presynaptic stimulation followed by postsynaptic depolarization) or converse pairing (depolarization followed by presynaptic stimulation). BTSP can be interpreted as a product of eligibility trace, an input-specific signal lasting for a few seconds, and instructive signals by postsynaptic depolarization, which induces synaptic potentiation in eligible synapses. For converse BTSP (cBTSP), the instructive signal must also influence the synapse-specific signal over a few seconds. Studies suggest that BTSP requires a plateau potential and Ca^2+^ spikes in dendrites, perhaps providing instructive signals^1,7^. However, the molecular representations of the eligibility trace and the instructive signal are unknown.

Using two-photon fluorescence lifetime imaging (2pFLIM) of a fluorescent resonance energy transfer (FRET) sensor, previous studies have shown that CaMKII is activated in a synapse-specific manner during glutamate uncaging-induced structural plasticity of dendritic spines^8,9^. CaMKII remains active for 1–6 s after glutamate uncaging owing to autophosphorylation at the T286 site^9,10^. This time scale of CaMKII activation (seconds) makes it a potential entity for the eligibility trace. However, a previous study suggests that CaMKII is required for BTSP expression but not induction^11^.

In this study, we investigated the role of CaMKII in BTSP using an improved CaMKII conformational sensor in hippocampal slices. First, we developed a protocol to induce BTSP at individual dendritic spines using glutamate uncaging. Second, we improved the sensitivity of a CaMKII sensor by about twofold. Using this new sensor, instead of synapse-specific CaMKII activity, we observed DDSC tens of seconds after the induction of BTSP. We confirmed the requirement of DDSC for BTSP by optogenetically inhibiting CaMKII^12^. Finally, we showed that DDSC and BTSP require IP_3_-dependent Ca^2+^ release from internal stores. Our experiments demonstrate a crucial role of non-synapse-specific CaMKII activation as an instructive signal that spans an extended time scale (tens of seconds) in BTSP.

## BTSP can be induced in single spines

**Fig. 1:**
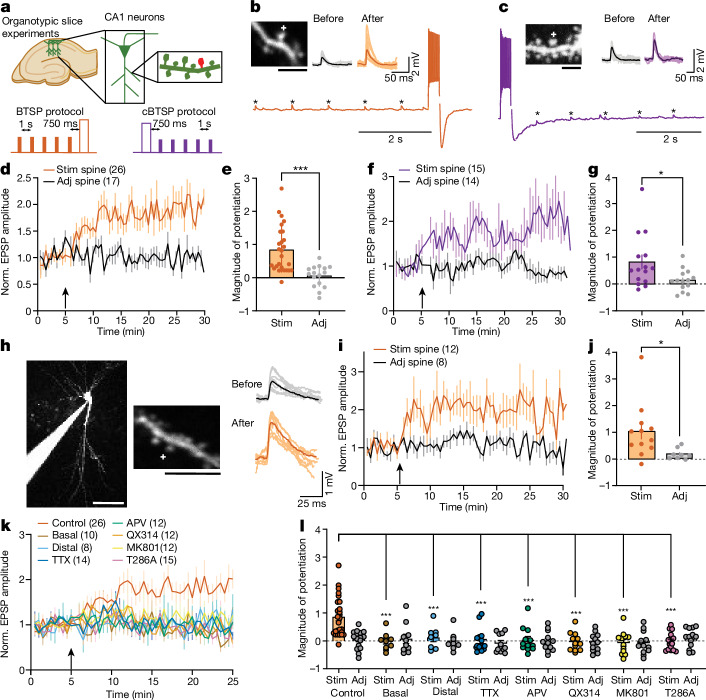
BTSP in single dendritic spines with glutamate uncaging. **a**, Top, schematic of the experimental set up. Bottom, BTSP and cBTSP protocols: 5 uncaging pulses were given at a spine before (orange) or after (purple) a 600 pA current injection (300 ms width) with a 750 ms delay. **b**, Representative image of a CA1 dendrite in an organotypic hippocampal slice (top left), uncaging-evoked EPSPs (ten recordings and average) before and after BTSP from the spine (top right) and electrical trace during BTSP protocol (bottom). **c**, Same as **b**, but with the cBTSP protocol. **d**,**e**, Averaged time course (**d**) and summary of magnitude of potentiation (25–30 min) (**e**) of uncaging-evoked normalized (Norm.) EPSP amplitudes (0–5 min) at stimulated (Stim) (*n* = 26) and adjacent (Adj) spines (*n* = 17) in response to BTSP (arrow). Two-tailed unpaired *t*-test, (*t*_40_ = 4.293, *P* = 0.0001). **f**,**g**, Same as **d** and **e**, but with the cBTSP protocol (stimulated spines, *n* = 15; adjacent spines, *n* = 14). Two-tailed unpaired *t*-test, (*t*_27_ = 2.366, *P* = 0.025). **h**, Left, representative Alexa-594 filled image of a CA1 neuron and dendritic shaft in acute hippocampal slices, in which BTSP was induced. Right, uncaging-evoked EPSP traces of stimulated spine (ten recordings and average) before and after BTSP induction. **i**,**j**, Same as **d** and **e**, but with the BTSP protocol in acute hippocampal slices (stimulated spines, *n* = 12; adjacent spines, *n* = 8). Two-tailed unpaired *t*-test (*t*_18_ = 2.276, *P* = 0.0353). **k**,**l**, Same as **d** and **e**, but with the BTSP protocol under various conditions (basal, *n* = 10; distal, *n* = 8; TTX stimulated, *n* = 14, adjacent, *n* = 11; APV, *n* = 12; QX314, *n* = 12; MK801, *n* = 12; T286A stimulated, *n* = 15; adjacent, *n* = 14). Two-way analysis of variance (ANOVA) (*F*_17,176_ = 4.395, *P* = 0.00000016) with Dunnett’s multiple comparison test (all *P* values are shown in the source data file). The data in **d**–**g** and **i**–**l** are presented as the mean ± s.e.m. The plus symbol in images depicts a BTSP-stimulated spine. *P < 0.05, ***P < 0.001. Scale bars, 2.5 μm (**b**,**c**), 5 μm (**h**, right) or 50 μm (**h**, left).

To investigate whether BTSP can be induced in single spines, we used whole-cell patch-clamp electrophysiology on CA1 neurons in organotypic hippocampal slices. We measured two-photon uncaging-evoked excitatory postsynaptic potentials (EPSPs) from one to two spines on secondary branches of proximal apical dendrites before and after the induction of BTSP (Fig. 1a, orange). To induce BTSP, we delivered a train of 5 uncaging pulses on 1 spine at 1 Hz intervals, and then after a 750 ms delay from the last pulse, we gave a current injection (600 pA, 300 ms) (Fig. 1b). This protocol induced a 90 ± 14% (*n* = 26) potentiation in EPSP amplitude in the stimulated spines (Fig. 1d,e), but not in adjacent spines (1 ± 9%, *n* = 17; Fig. 1d,e). In the cBTSP protocol (Fig. 1a, purple), we applied a current injection (600 pA, 300 ms) that was delivered 750 ms before the first pulse of 5 uncaging pulses at 1 Hz (Fig. 1c). This protocol also induced a similar potentiation (81 ± 25%, *n* = 15) in EPSP amplitude in stimulated spines but not in adjacent spines (13 ± 10%, *n* = 14) (Fig. 1f,g). Similarly, in acute hippocampal slices (from mice aged postnatal day 25 (P25)–P35), the BTSP protocol induced potentiation (103 ± 29%, *n* = 12) in stimulated spines but not in adjacent spines (18 ± 7%, *n* = 8) (Fig. 1h–j). These results demonstrate that BTSP can be induced in single dendritic spines in a synapse-specific manner both in acute and organotypic slices.

**Extended Data Fig. 1:**
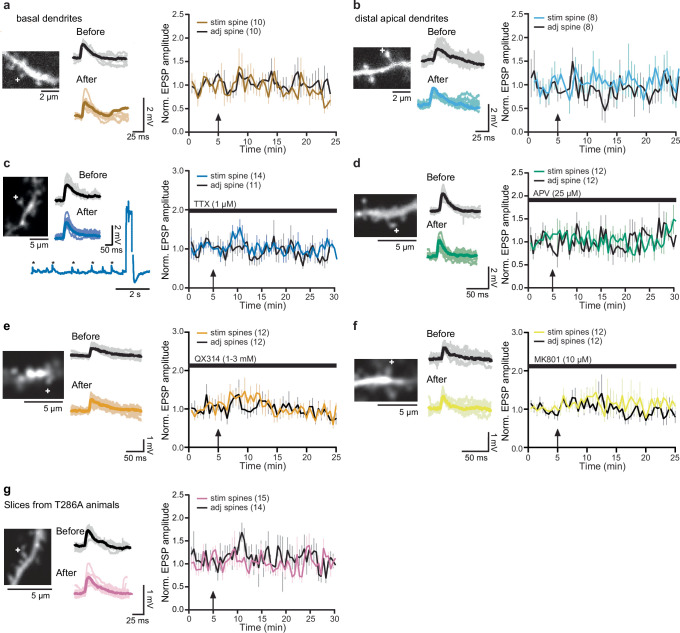
BTSP protocol fails to induce plasticity in basal or distal synapses and is dependent on spiking, NMDAR and CaMKII. **a**, Left: Representative basal dendritic image where BTSP was induced in one spine (+); Middle: Raw traces of EPSPs in a stimulated spine (10 recordings and average) before and after BTSP induction. Right: Averaged time course of normalized EPSP amplitude following BTSP induction in basal dendrites in stimulated and adjacent spines. Arrow depicts BTSP induction. **b**, Same as **(a)**, but in distal dendrites. **c**, Left: Representative image of an apical dendritic shaft, where BTSP protocol was induced in one spine (+) in the presence of TTX (1 µM). All experiments were performed after incubating the slices for ~30 mins in TTX. Middle: Raw traces of EPSP in the stimulated spine (10 recordings and average) in TTX. Right: Averaged time course of normalized EPSP amplitude in TTX in stimulated and adjacent spines. **d**, Same as **(c)**, but performed after incubating the slices for 30 mins in the presence of APV (25 µM). **e**, Same as **(c)**, but the experiments were performed in the presence of QX314 (1-3 mM) in the patch pipette. **f**, Same as **(c)**, but performed after incubating the slices for 30 mins in the presence of MK801 (10 µM). **g**, Same as **(c)**, but in hippocampal slices made from *Camk2a*^T286A^ mice. The number of cells is mentioned in the figure panels in parentheses.

Because basal and distal dendrites receive different inputs as compared to proximal apical dendrites^13^, we investigated whether BTSP can also be induced in these dendrites. Notably, the same BTSP protocol failed to induce EPSP potentiation at basal or distal apical dendrites (>200 μm from the soma) (basal, 7 ± 17%, *n* = 10; distal, 12 ± 12%, *n* = 8) (Fig. 1k,l and Extended Data Fig. 1a,b). Thus, the BTSP induction mechanism may depend on the location of dendritic branches. Next, we examined the molecular mechanism of BTSP using pharmacological inhibitors and transgenic mice. The voltage-gated sodium channel inhibitor tetrodotoxin (TTX; 1 μM) and the NMDAR inhibitor APV (50 μM) inhibited BTSP induction in stimulated spines (Fig. 1k,l and Extended Data Fig. 1c,d). BTSP was also inhibited by QX314 (1–3 mM), a membrane-impermeable sodium channel blocker, included in the patch pipette (Fig. 1k,l and Extended Data Fig. 1e). As the BTSP protocol should not effectively release Mg^2+^ block from NMDARs, we further investigated a potential role for non-ionotropic NMDAR signalling using MK-801, a channel pore blocker for NMDAR^14,15^. MK-801 also blocked BTSP, which suggested that canonical ionotropic NMDAR signalling is required for BTSP (Fig. 1k,l and Extended Data Fig. 1f). Furthermore, similar to a previous study^11^, mutant mice in which the Ca^2+^-independent autonomous activity of CaMKIIα generated by T286 autophosphorylation is prevented (*Camk2a*^T286A^) showed no BTSP (Fig. 1k,l and Extended Data Fig. 1g). These results suggest that similar to Hebbian long-term potentiation (LTP), postsynaptic spiking, activation of NMDAR and generation of autonomous CaMKII activity are required for BTSP induction.

## Dendritic, delayed, stochastic CaMKII activity

**Fig. 2:**
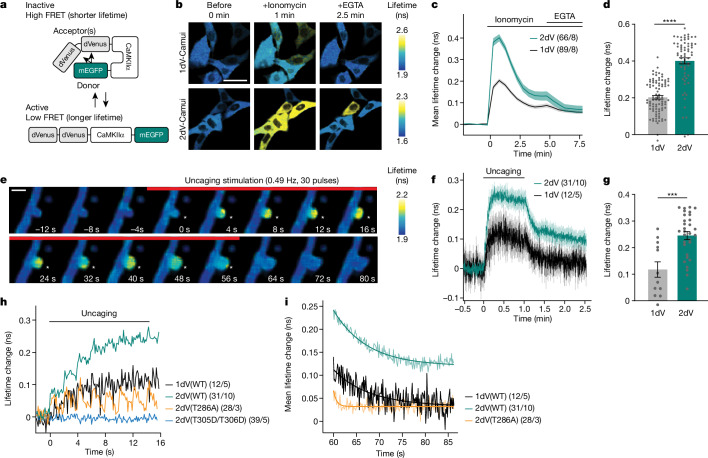
Optimization and characterization of a new conformational CaMKII FRET sensor. **a**, Schematic of the improved CaMKIIα sensor 2dV-Camui. mEGFP, monomeric EGFP. **b**, Representative fluorescence lifetime images of HeLa cells expressing 2dV-Camui (bottom) or original Green Camuiα (1dV-Camui) (top) before, during ionomycin and during EGTA. **c**,**d**, Mean time course (**c**) and summary of the peak (1 min after 3 µM ionomycin application) (**d**) of fluorescent lifetime changes of 2dV-Camui (2dV) (*n* = 89 cells over 8 independent experiments) and Green Camuiα (1dV) (*n* = 66 cells, 8 experiments) in HeLa cells. 2dV showed around twofold higher sensitivity (two-tailed unpaired *t*-test, (*t*_153_ = 11.01, *P* = 2.0 × 10^–21^) (**d**). **e**, Representative fluorescence lifetime images of CA1 dendrites in hippocampal culture before, during and after glutamate uncaging at 0.49 Hz in zero extracellular Mg^2+^. Asterisk depicts the spine where glutamate was uncaged. Similar activation profiles were observed in more than 95% of stimulated spines over 8 neurons. **f**,**g**, Mean time course (**f**) and summary of the peak (6th–11th uncaging pulses) (**g**) of fluorescence lifetime changes in stimulated spines. 2dV (*n* = 31 spines, 10 neurons) showed higher sensitivity than 1dV (*n* = 12 spines, 5 neurons). Two-tailed unpaired *t*-test, (*t*_41_ = 4.26, *P* = 1.18 × 10^–4^) (**g**). **h**,**i**, Closer views of mean lifetime changes at spines during the first 8 uncaging pulses (**h**) and the decay kinetics after the last uncaging pulse (**i**) in wild-type 1dV(WT) (*n* = 12 spines, 5 neurons), 2dV(WT) (*n* = 31 spines, 10 neurons), 2dV(T286A) (*n* = 28 spines, 3 neurons) and 2dV(T305D/T306D) (*n* = 39 spines, 5 neurons). The fitting curves indicate single exponential fitting (*y* = Aexp(–*t*/*τ*) + B, where the fast time constant (*τ*) is 7.9, 7.3 and 0.7 s for 1dV(WT), 2dV(WT) and 2dV(T286A), respectively. The data are presented as the mean ± s.e.m. ****P < 0.0001. Scale bars, 2 μm (**e**) or 50 μm (**b**).

**Extended Data Fig. 2:**
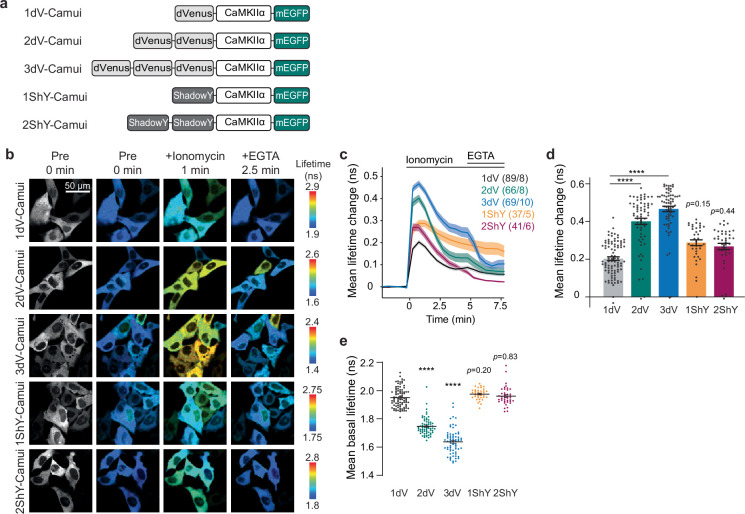
Characterization of novel conformational CaMKII FRET sensor. **a**, A schematic of screened CaMKII sensors where N- and C-termini of CaMKIIα are labelled with 1-3 dimVenus or 1-2 ShadowY acceptor(s) and donor EGFP fluorophores. **b**, Grayscale and fluorescence lifetime images of HeLa cells expressing each Camuiα sensor variant. Each image is before (pre), 1 min after ionomycin (3 µM) and 2.5 min after EGTA (8 mM) application. **c-d**, Averaged time course of lifetime change in HeLa cells expressing Camuiα sensor variants and the peak of fluorescent lifetime change in response to bath application of ionomycin (1 min post-ionomycin application) shown in c. (n =# of cells/# of independent experiments: 1dV, n = 89/8; 2dV, n = 66/8; 3dV, n = 69/10; 1ShY, n = 37/5; 2ShY, n = 41/6). One-way ANOVA (F(4, 297) = 68.1) followed by Dunnett’s post hoc test (1dV vs. 2dV, p = 7.3e-13; 1dV vs. 3dV, p = 7.3e-13, 1dV vs. 1ShY, p = 4.2e-4; 1dV vs. 2ShY, p = 7.5e-3) **(d)**. The data are presented as mean ± s.e.m. **e**, Averaged basal lifetime of each CaMKII sensor shown in c (n same as **d**). Multiplying dimVenus acceptors showed significantly lower basal lifetimes, indicating higher FRET efficiency. One-way ANOVA (F(4, 297) = 296.1) followed by Dunnett’s post hoc test (1dV vs. 2dV, p = 7.3e-13; 1dV vs. 3dV, p = 7.3e-13, 1dV vs. 1ShY, p = 0.20; 1dV vs. 2ShY, p = 0.83). The data are presented as mean ± s.e.m. **p < 0.01, ****p < 0.0001.

**Extended Data Fig. 3:**
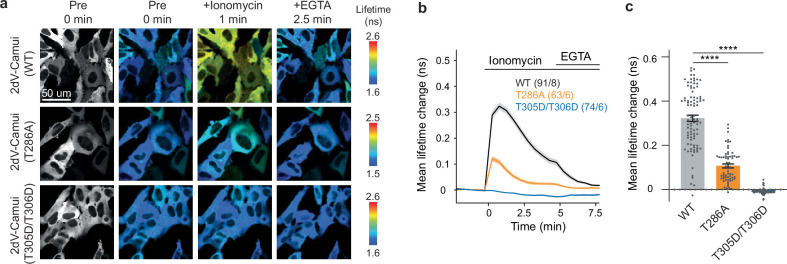
Validation of 2dV-Camui conformational CaMKII FRET sensor with wildtype, T286A or T305D/T306D CaMKII mutants. **a**, Grayscale and fluorescence lifetime images of HeLa cells expressing 2dV-Camuiα sensors, which have wildtype CaMKII (WT), CaMKII mutant deficient autonomous activation (T286A), or CaMKII mutant deficient in Ca2 + /calmodulin binding (T305/T306D). Each image is before (pre), 1 min after ionomycin (3 µM) and 2.5 min after EGTA (8 mM) application. **b, c**, Averaged time course (**b**) and summary of the peak (1 min post-ionomycin application) (**c**) of fluorescent lifetime changes of 2dV-Camui in WT and mutant CaMKII expressing HeLa cells in response to bath application of ionomycin. The data are presented as mean ± s.e.m. (n = # of cells/# of independent experiments: WT, n = 91/8; T286, n = 63/6; T305D/T306D, n = 74/6). c, One-way ANOVA (F(2, 225) = 265.5) followed by Dunnett’s post hoc test (WT vs. T286A, p = 4.0e-13; WT vs. T305D/T306D, p = 4.0e-13). ****p < 0.0001.

**Extended Data Fig. 4:**
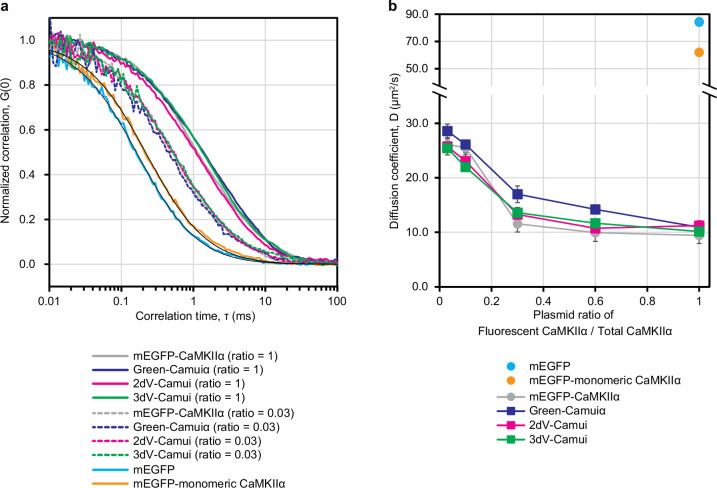
Fluctuation correlation spectroscopy (FCS) analysis of CaMKII sensors. **a**, Typical normalized correlation curves obtained from 2-photon TCSPC (Time-Correlated Single Photon Counting) FCS for the indicated samples in HEK293FT cell lysate. To investigate whether the sensors were co-polymerised with nonlabelled CaMKIIα, the cells were co-transfected with non-labelled CaMKIIα at the indicated plasmid ratios. The black line shows fit curves of a correlation function given by: $G(\tau )=G(0){(1+\tau /{\tau }_{xy})}^{-1}{(1+{({w}_{xy}/{w}_{z})}^{2}\tau /{\tau }_{xy})}^{-0.5}$ where *G*(0) is the correlation at time 0, *τ* is the correlation time, *w*_xy_ is the lateral and *w*_z_ the axial 1/*e*^2^-radii of the 2-photon excitation volume, measured as 0.34 µm and 1.49 µm, respectively, by scanning 0.1-µm fluorescent bead. The diffusion coefficient $D={w}_{{xy}}^{2}/8{\tau }_{{xy}}$ was determined from the average lateral diffusion time *τ*_xy_ obtained by curve fitting. **b**, Diffusion constants of the sensors or mEGFP-CaMKIIα co-expressed with non-labelled CaMKIIα at various plasmid ratios. The diffusion coefficients (ratio = 1) were 9.4 ± 1.5 µm^2^/s (n = 15 measurements) for mEGFP-CaMKIIα, 11 ± 0.6 µm^2^/s (n = 6) for Green-Camuiα, 11 ± 0.9 µm^2^/s (n = 7) for 2dV-Camui, 10 ± 0.9 µm^2^/s (n = 6) for 3dV-Camui, 84 ± 2.9 μm^2^/s (n = 17) for EGFP, and 62 ± 2.0 μm^2^/s (n = 8) for monomeric CaMKIIα (truncated CaMKIIα [1-306]), respectively. The data are indicated as mean ± SD.

**Extended Data Fig. 5:**
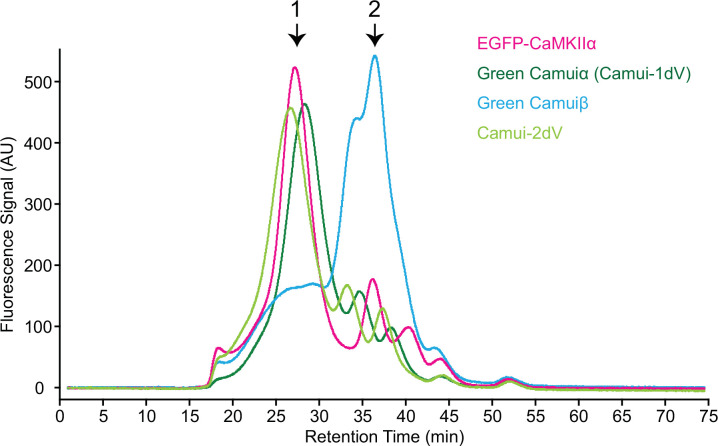
Chromatography assay of CaMKII sensors. To estimate the approximate oligomeric state of CaMKII sensors, we performed FSEC on EGFP-CaMKIIα and various Camui proteins expressed in HEK293 cells by transient transfection. The cell lysates expressing these proteins were directly injected to a Superose-6 column, and EGFP fluorescence was detected using 475/507 nm excitation/emission wavelengths. Green Camuiα (1dV-Camui), Green Camuiβ (in which CaMKIIβ subunit is used instead of CaMKIIα), and 2dV-Camui are compared with EGFP-CaMKIIα. 2dV-Camui showed a similar retention time to EGFP-CaMKIIα and 1dV-Camui (peak at ~27 min; arrow 1), suggesting the formation of a dodecamer. However, Green Camuiβ showed a high fraction of lower oligomeric species represented by a peak at ~35 min (arrow 2).

If CaMKII represents the eligibility trace, its activation should be localized in the stimulated spines and last for a few seconds. This synaptic input will not release the Mg^2+^ block; thus, Ca^2+^ signalling is likely to be small. To detect anticipated small levels of CaMKII activity during BTSP, we optimized our CaMKII sensor by putting two accepters in the original Green Camuiα sensor^8^, which produced 2dV-Camui (Fig. 2a and Supplementary Information). 2dV-Camui displayed a twofold higher signal with a similar time course compared with the unmodified version when tested in cell lines (Fig. 2b–d and Extended Data Fig. 2) and in uncaging-evoked CaMKII activation in dendritic spines. Note that structural LTP experiments in Fig. 2 are under zero extracellular Mg^2+^ conditions. All other experiments were performed using 1 mM Mg^2+^ (ref. ^8^) (Fig. 2e–g). As expected, we observed a rapid decay in CaMKII activation for a sensor in which the crucial autophosphorylation site (T286) is mutated^10^ (Fig. 2h,i). Also, mutations that turn off calmodulin binding (T305 and T306) eliminated its activation (Fig. 2h and Extended Data Fig. 3). Moreover, we confirmed that this sensor forms the usual multimeric holoenzyme based on fluctuation correlation spectroscopy and fluorescence-coupled size-exclusion chromatography (Extended Data Figs. 4 and 5).

**Fig. 3:**
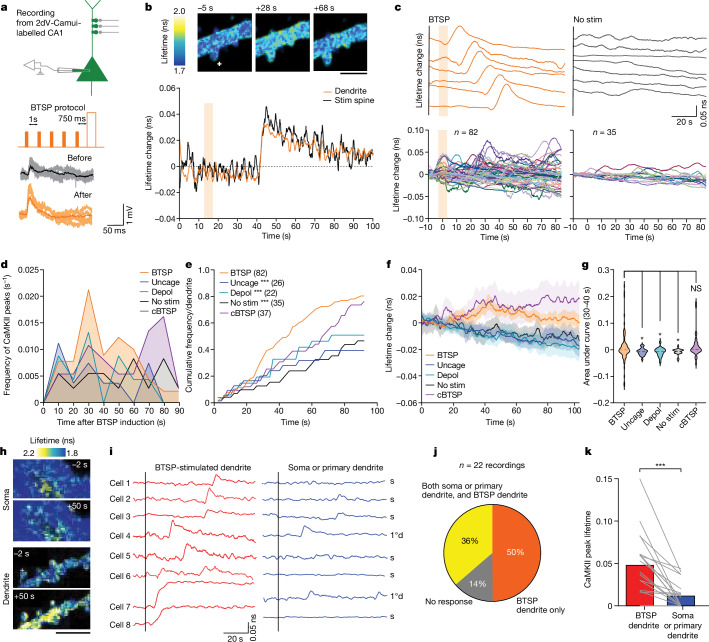
DDSC after BTSP induction. **a**, Experimental set up (top), BTSP protocol (middle) and raw traces of EPSPs (ten recordings and average) before and after BTSP protocol (bottom). **b**, Fluorescence lifetime images (top) and time course (bottom) of a dendritic segment expressing 2dV-Camui during BTSP induction (shaded region, BTSP protocol). **c**, Representative traces (top) and all dendrites (bottom) for which fluorescence lifetime changes of 2dV-Camui were measured after BTSP (left) and no stimulation (no stim, right). Shaded region, BTSP protocol. **d**, Frequency of DDSC onsets after BTSP and cBTSP induction compared with uncaging only (uncage), current injection only (depol) and no stimulation controls. **e**, Cumulative frequency of **d**. Two-sided Kolmogorov–Smirnov test with Bonferroni’s correction, *P* = 0.00032 for BTSP versus uncage; *P* = 0.0000072 for BTSP versus depol; *P* = 0.00000026 for BTSP versus no stim; and *P* = 0.12 for BTSP versus cBTSP. **f**,**g**, Averaged time course (**f**) and area under the curve (30–40 s after BTSP) (**g**) of lifetime change of CaMKII activity under all five conditions. Data are presented as the mean ± s.e.m. in **f** and median and second quartile along with individual values in **g**. Two-way ANOVA (*F*_4,100_ = 5.434, *P* = 0.0005) with Dunnett’s multiple comparison test (all *P* values are shown in the source data file). NS, not significant. **h**, Fluorescence lifetime images of 2dV lifetime changes before (−2 s) and after (+50 s) BTSP protocol at secondary dendrites or soma. **i**, Representative 2dV-Camui traces of dendrite and of soma or primary dendrites from eight different cells. If the soma was not in the same *z* plane as the stimulated dendrite, the primary dendrite was used. **j**, Pie chart showing percentage of recordings that showed an increase in CaMKII activity either in both stimulated dendrite and soma (or primary dendrite) or specifically in the dendrites. **k**, Amplitude of DDSC in BTSP-induced dendrites and in soma or primary dendrites. The data are presented as the mean and individual values. Two-tailed paired *t*-test (*t*_19_ = 4.924, *P* = 0.000094). Plus symbol in images depicts the BTSP-stimulated spine. Number of dendrites are mentioned in the figure panels in parentheses wherever appropriate. *P < 0.05, ***P < 0.001. Scale bars, 2.5 μm (**b**) or 5 μm (**h**).

**Extended Data Fig. 6:**
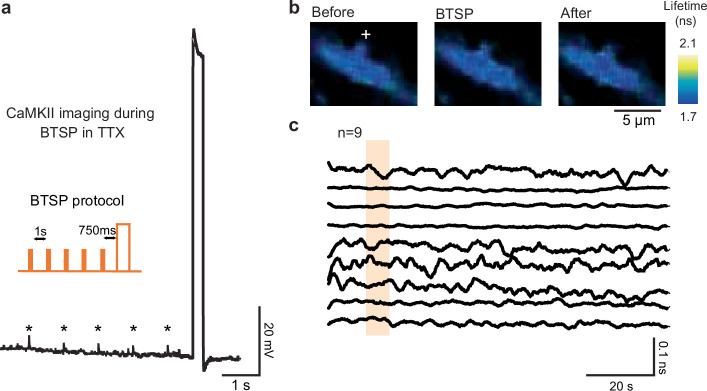
CaMKII activity is abolished in the presence of TTX. **a**, Representative recording during BTSP showing response to 5 uncaging pulses and no action potential during the current injection. **b**, Fluorescence lifetime images of 2dV-Camui expressing dendrite before, during and after the BTSP (BTSP) protocol in TTX (1 µM). “+” depicts the BTSP stimulated spine. **c**, Individual dendritic lifetime measurements of 2dV-Camui in TTX before during and after BTSP (shaded region, BTSP protocol). The number of dendrites is mentioned in the figure panel.

**Extended Data Fig. 7:**
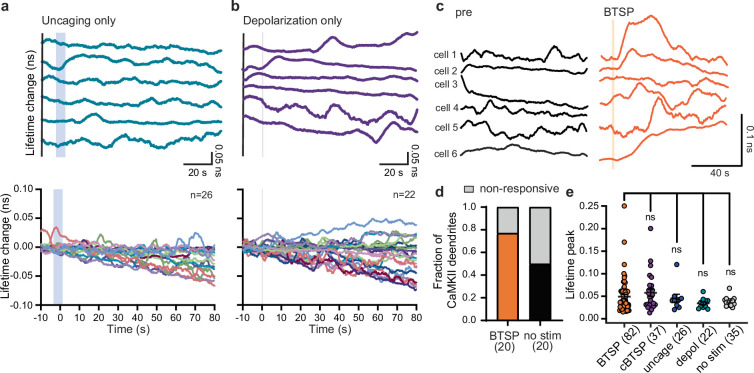
Control experiments show that delayed dendritic CaMKII is specific to BTSP induction. **a-b**, Top: Representative traces of CaMKII dendritic activity (smoothened by moving average of 60 points, 7.8 frames per second) of uncaging only and depolarization only experiments. Bottom: Lifetime change of all dendritic CaMKII activity for uncaging only and depolarization only. (shaded region, uncaging or depolarization protocol). **c**, Representative traces of smoothened 2dv-Camui dendritic recording from the same cells before (black) and after (orange) the BTSP protocol. (shaded region, BTSP protocol). **d**, Fraction of dendrites showing CaMKII activity in pre (no stim, n = 20) versus after BTSP (n = 20) induction. **e**, Peak amplitude of CaMKII activation in responsive BTSP (0.048 ± 0.005 ns, n = 82) and converse BTSP (0.057 ± 0.007 ns, n = 37) dendrites as compared to control dendrites- no-stim (0.038 ± 0.002 ns, n = 35), uncaging only (0.045 ± 0.008 ns, n = 26), and depolarization only (0.034 ± 0.003 ns, n = 22)). The data are presented as mean ± s.e.m. One-way ANOVA (F(4,121) = 1.223) followed by Dunnett’s post hoc test, BTSP vs cBTSP p = 0.73; BTSP vs uncage p = 0.99; BTSP vs depol p = 0.56; BTSP vs no stim p = 0.77. The number of dendrites is mentioned in the figure panels.

**Extended Data Fig. 8:**
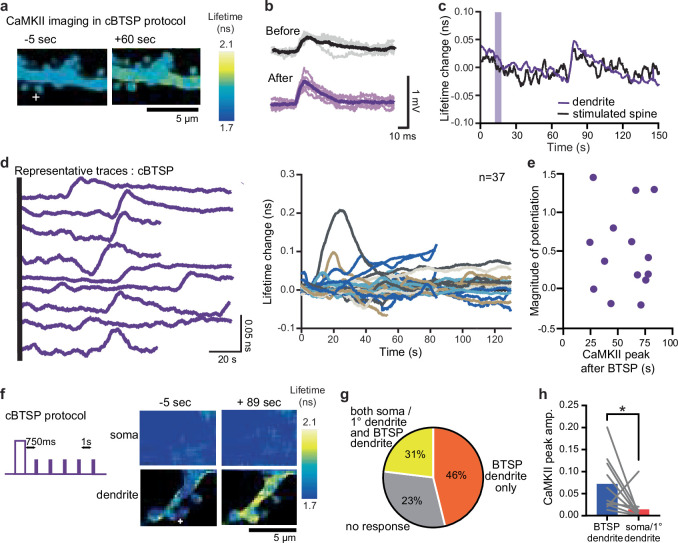
Converse BTSP protocol induces delayed dendritic and stochastic CaMKII (DDSC). **a**, Fluorescence lifetime images of 2dV-Camui expressing dendrite before, during and after the converse BTSP (cBTSP) protocol. “+” depicts the cBTSP stimulated spine. **b**, Representative EPSP traces from stimulated spines (10 recordings and average) before and after the cBTSP protocol. **c**, Time course of fluorescence lifetime changes in the stimulated spine and nearby dendrite from the dendrite in **(b)** (filtered) (shaded region, cBTSP protocol). **d**, Left: Representative dendritic 2dV-Camui recordings. Right: All 2dV-Camui recordings (filtered) during cBTSP induction. **e**, Relationship between the magnitude of potentiation and time of the first CaMKII peak after cBTSP protocol in a subset of neurons we could measure synaptic potentiation (Pearson R correlation, n = 14 pairs, r^2^ = 0.02, p = 0.6). **f**, Simultaneous FLIM imaging of 2dV-Camui in soma and stimulated dendrite during cBTSP. **g**, Pie chart for 13 recordings, out of which 46% showed an increase in CaMKII activity in stimulated dendrites but not in the soma or primary dendrites. 31% of the recordings showed an increase in CaMKII activity in both the stimulated dendrite and the soma/primary dendrite, and 23% of the dendrites showed no CaMKII activity. **h**, Peak amplitude of CaMKII activation events in the stimulated dendrite compared with that in the soma or the primary dendrite. The data are presented as mean and individual values. Paired two-tailed t-test (t(9) = 2.35, p = 0.04). The number of dendrites are mentioned in the figure panels. *p < 0.05.

**Extended Data Fig. 9:**
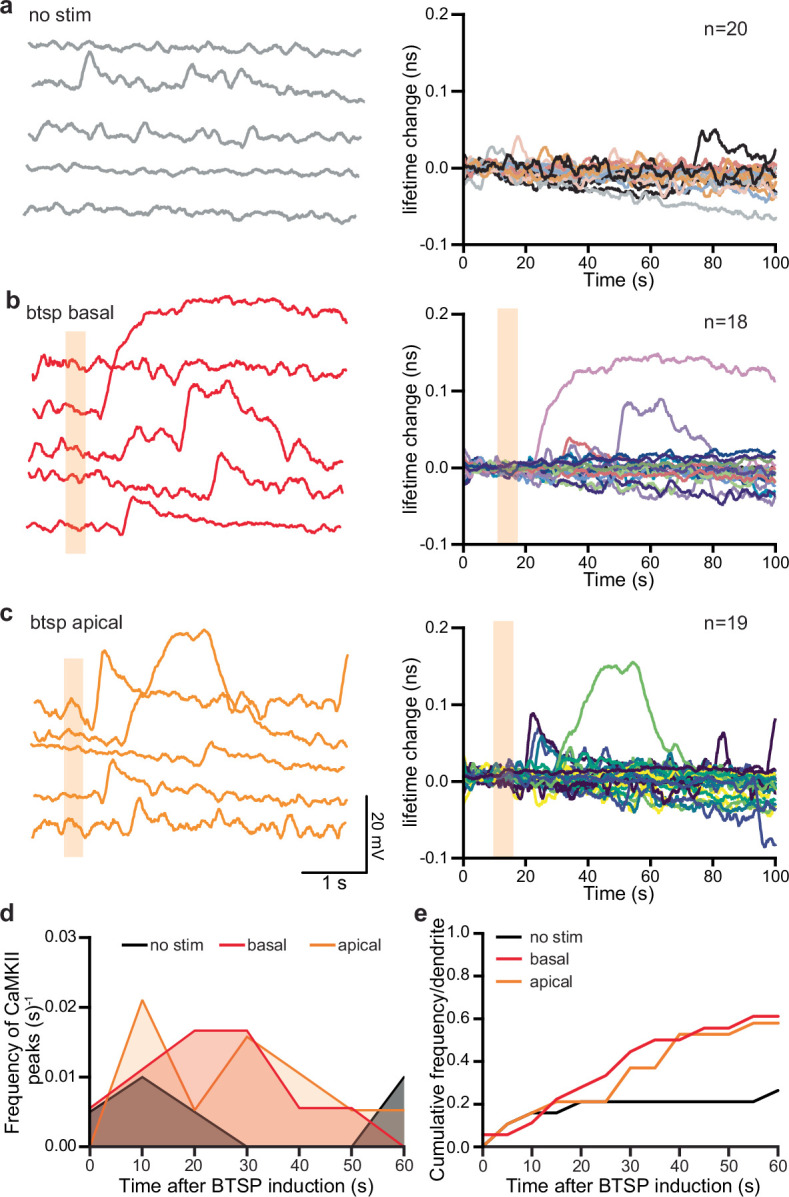
Similar DDSC is observed in basal dendrites. **a**, Left: Representative dendritic 2dV-Camui recordings in basal dendrites before induction of BTSP. Right: All 2dV-Camui recordings (filtered) before BTSP induction. **b**, Same as **(a)**, but performed after BTSP induction (shaded region, BTSP protocol). **c**, Same as **(a)**, but performed in corresponding proximal apical dendrites after BTSP induction (shaded region, BTSP protocol). **d**, Frequency of DDSC onsets after BTSP induction in apical (orange) and basal dendrites (red) compared with no stimulation (black) controls. **e**, Cumulative frequency of **(d)**. Two-sided Kolmogorov-Smirnov test with Boenhoffer’s correction, basal vs apical p = 1.0; basal vs no stim p = 0.018. The number of dendrites is mentioned in the figure panels. *p < 0.05.

To investigate CaMKII activity during BTSP, we performed whole-cell patch-clamp electrophysiology on 2dV-Camui-expressing CA1 neurons in organotypic slices and imaged CaMKII activity during BTSP induction (Fig. 3a). Although there was no detectable CaMKII activity during BTSP induction (Fig. 3b–d), we observed CaMKII activation both in stimulated and adjacent dendrites with a delay of tens of seconds (Fig. 3b,c). This delayed global CaMKII activity showed stochasticity in its timing. Overall, 79% of dendrites showed delayed CaMKII activation, with peak timings ranging from 0 to 100 s and particularly clustered around 30–40 s (*n* = 82 dendrites; Fig. 3c–e). This CaMKII activity was abolished by TTX, which suggested that postsynaptic firing or ongoing circuit activity is involved in this process (*n* = 9; Extended Data Fig. 6). In control neurons without any stimulation, we also observed CaMKII activation but with a significantly lower frequency (no stimulation, *n* = 35; Fig. 3c–e). Similarly, uncaging pulses without current injection (uncaging, *n* = 26; Extended Data Fig. 7a) and current injection without uncaging (depolarization, *n*  = 22; Extended Data Fig. 7b) showed CaMKII activation with a frequency substantially lower than in BTSP-induced dendrites. Similar delayed CaMKII activity was observed when the cBTSP protocol was used, but the timing of CaMKII activation was more delayed (*n* = 37, 70–100 s; Fig. 3c–e and Extended Data Fig. 8a–e). In a subset of experiments for which we recorded CaMKII activation before BTSP induction for a longer time (100 s) and after BTSP, we also observed a significant increase in CaMKII activation after BTSP induction (Extended Data Fig. 7c,d). The average time course (Fig. 3f) showed a downwards drift in the measurement, perhaps due to photobleaching. However, on top of the drift, there was a substantial increase in CaMKII activity in BTSP-induced and in cBTSP-induced dendrites about 30–40 s after the protocol, but not in the control groups (uncaging, depolarization and no stimulus). The area under the curve in BTSP and cBTSP dendrites from 30 to 40 s showed significantly higher CaMKII activation than with the three controls (Fig. 3g). We found no difference in the peak amplitude of CaMKII activity between BTSP-induced and cBTSP-induced samples compared with the three controls (Extended Data Fig. 7e), which indicated that BTSP increases the frequency, but not the amplitude, of CaMKII activation. Similar CaMKII activation was also observed in basal dendrites, although BTSP was not induced in these dendrites (Extended Data Fig. 9).

**Extended Data Fig. 10:**
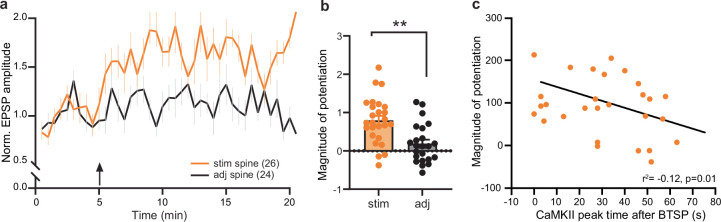
BTSP-induced synaptic potentiation is induced in 2dV-Camui labeled neurons. **a-b**, Averaged time course **(a)** and summary (25-30 min) of normalized EPSP amplitude **(b)** in neurons expressing 2dV-Camui in the stimulated (n = 26) and adjacent (n = 24) spines. The data are presented as mean ± s.e.m. Two-tailed unpaired t-test (t(40) = 3.17, p = 0.0013). The arrow depicts BTSP induction. **c**, Correlation between the magnitude of potentiation and time of CaMKII peak after the BTSP protocol (Two-sided Pearson R correlation, n = 28 pairs, r^2^ = −0.12, p = 0.01). The number of cells is mentioned in the figure panels. ***p < 0.01.

In a subset of the above experiments, we recorded EPSPs in stimulated spines and found similar BTSP potentiation in the CaMKII-labelled neurons (Extended Data Fig. 10a,b). Moreover, there was an inverse correlation between the magnitude of potentiation and the time of CaMKII occurrence after BTSP induction (Extended Data Fig. 10c), which suggested that earlier CaMKII activity tends to result in a higher magnitude of potentiation. This correlation was not observed for cBTSP (Extended Data Fig. 8e). These experiments suggest that both the BTSP and the cBTSP protocols induce DDSC. Moreover, DDSC required both presynaptic and postsynaptic components, which indicated that CaMKII upstream signalling can integrate these components.

To investigate the spatial profile of DDSC, we imaged CaMKII activity simultaneously in stimulated dendrites and in the soma or primary dendrites following BTSP induction (Fig. 3h). DDSC was more predominant in the BTSP-induced dendrites than in the soma or primary dendrites (Fig. 3i–k). Moreover, 50% of the recordings showed CaMKII activity specific to the BTSP-stimulated dendrites, whereas 36% of them showed CaMKII activity both in the BTSP-stimulated dendrites and in the soma (or primary dendrite) (Fig. 3j). Furthermore, the peak CaMKII amplitude in dendrites was significantly higher in the BTSP-induced dendrites than in the soma or primary dendrites (Fig. 3k). A similar spatial profile of DDSC was observed following the cBTSP protocol (*n* = 14; Extended Data Fig. 8f–h). Overall, these experiments suggest that DDSC has a limited spread to primary dendrites and the soma.

## DDSC coincides with Ca^2+^ plateau potentials

**Fig. 4:**
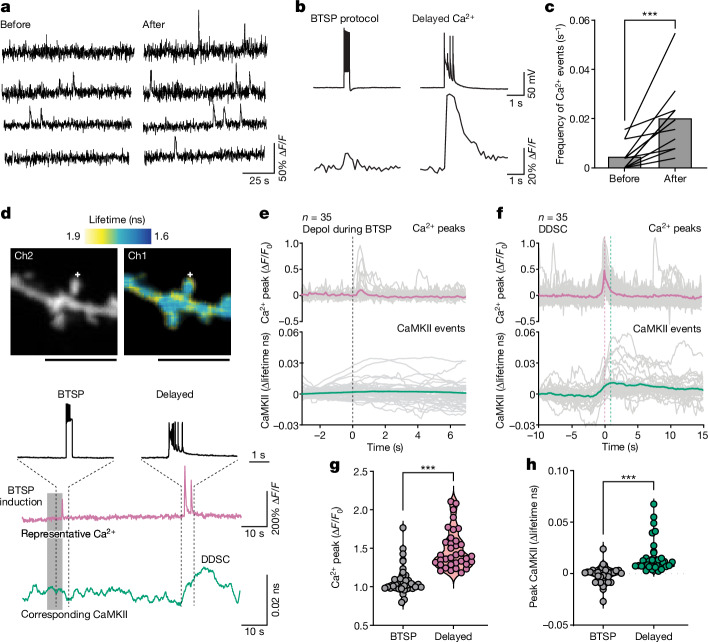
Ca^2+^ increases are associated with DDSC. **a**, Snippet of Ca^2+^ traces before (4 min) and after (4 min) BTSP induction in neurons filled with Cal-590 (50 µM) through a patch pipette. **b**, Representative traces of voltage recordings and corresponding Ca^2+^ traces. **c**, Frequency of Ca^2+^ events before and after BTSP induction (*n* = 12 dendrites from 12 cells). The data are presented as the mean and individual values. Two-tailed paired *t*-test (*t*_11_ = 4.550, *P* = 0.0008). **d**, Top, representative fluorescence lifetime images of a dendrite during simultaneous Ca^2+^ (Cal-590, intensity, Ch2) and CaMKII (2dV-Camui, FLIM, Ch1) imaging. Plus symbol depicts the BTSP-stimulated spine. Bottom, representative voltage, Ca^2+^ and CaMKII traces. Scale bars, 5 μm. **e**, Average Ca^2+^ increase (red) and CaMKII activity (green) in the stimulated dendrite during the BTSP protocol. Only the depolarization component of BTSP is shown (at *t* = 0). There was no detectable CaMKII activation during the BTSP protocol. **f**, Average of delayed Ca^2+^ (red) and CaMKII (green) detected using Ca^2+^ following BTSP induction. Events are aligned to the peak of Ca^2+^ events. CaMKII activity (>0.01 ns, from –5 to 5 s) was detected in 57% (20 out 35) of the delayed calcium events. **g**,**h**, Summary of Ca^2+^ (**g**) and CaMKII (**h**) peak amplitudes observed during the BTSP protocol (as in **e**) and after BTSP induction (as in **f**). Data are presented as the median and second quartile along with individual values. Two-tailed unpaired *t*-test for Ca^2+^ (*t*_62_ = 7.66, *P* = 1.6 × 10^–10^) and for CaMKII (*t*_67_ = 5.7, *P* = 4.5 × 10^–7^). Number of cells or events are mentioned in the panels in parentheses wherever appropriate. ***P < 0.001.

**Extended Data Fig. 11:**
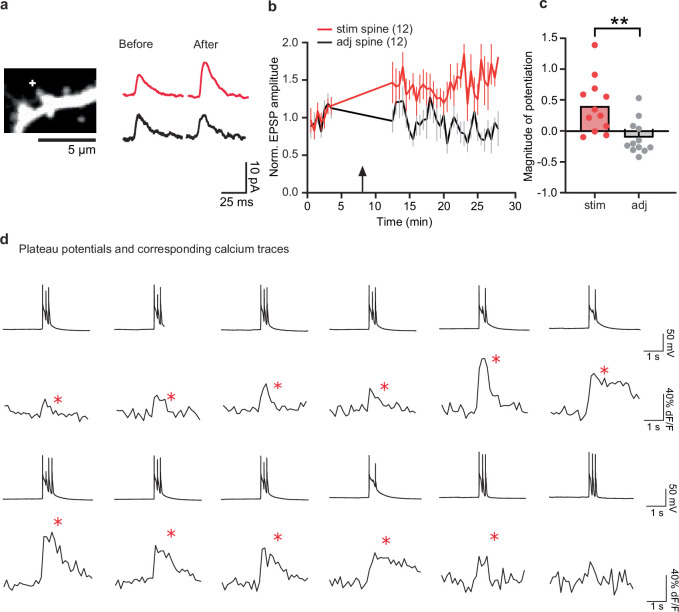
BTSP induces EPSP potentiation and plateau potentials observed during Ca^2+^ imaging experiments. **a**, Left: A representative dendrite of a neuron filled with Cal-590. “+” depicts the BTSP stimulated spine, Right: Average EPSP traces before and after BTSP protocol. **b**, Averaged time course of normalized EPSP amplitude in stimulated and adjacent spines. The gap in EPSP recording shows the time of calcium imaging (Fig. 4a–c). The arrow depicts BTSP induction. **c**, Group summary plot shows a higher magnitude of potentiation in stimulated spines than adjacent spines. The data are presented as mean and individual values. Two-tailed unpaired t-test (t(22) = 3.367, p = 0.003). **d**, Representative traces of voltage recordings showing that plateau potentials had corresponding Ca^2+^ traces (red *) in a majority of the examples (58/74, 3-standard deviation for Ca^2+^). The number of cells is mentioned in the figure panels. ***p < 0.01.

**Extended Data Fig. 12:**
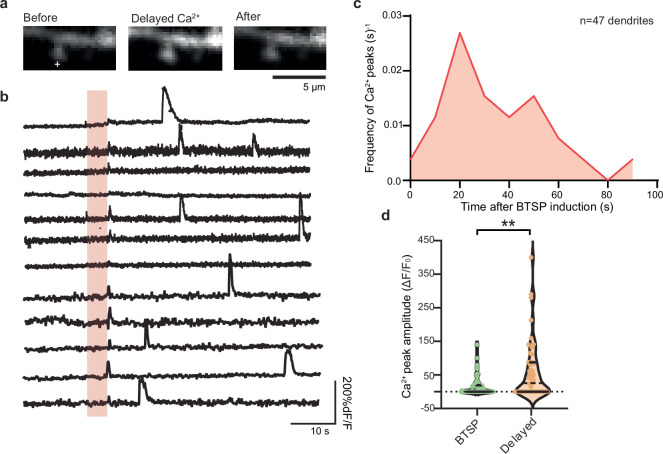
Characterization of delayed Ca^2+^ events in response to the BTSP protocol. **a**, Representative dendritic shaft filled with Cal-590 dye showing a Ca^2+^ event. “+” depicts the BTSP stimulated spine. **b**, Representative dendritic Ca^2+^ traces after the BTSP protocol (pink shaded). The traces show multiple Ca^2+^ events after BTSP, in addition to smaller event during current injection of BTSP protocol. **c**, The frequency of Ca^2+^ events as a function of the time after BTSP protocol. The frequency peak appears around ~20-30 secs after the BTSP protocol. **d**, Ca^2+^ peak amplitude showed a smaller Ca^2+^ during depolarization and a significantly larger delayed Ca^2+^ peak amplitude. Data are presented as median and second quartile, along with individual values. Two-tailed unpaired t-test (t(92) = 3.2, p = 0.002). The number of dendrites is mentioned in the figure panels. ***p < 0.01.

Because CaMKII activation requires an increase in Ca^2+^ levels^16^, we proposed that DDSC is associated with dendritic Ca^2+^ increases. To test this hypothesis, we performed Ca^2+^ imaging by filling the cell with Cal-590 (50–100 μM) dye in a whole-cell configuration for 4 min before and after the BTSP protocol (Fig. 4a–c). We confirmed that applying the BTSP protocol potentiated EPSPs in stimulated spines in dye-filled neurons (Extended Data Fig. 11a–c). Consistent with DDSC, most Ca^2+^ events occurred in close correlation with plateau potentials during 4-min recordings after the BTSP protocol (Fig. 4b and Extended Data Fig. 11d). The frequency of these delayed Ca^2+^ events peaked around 20–30 s, consistent with DDSC (Extended Data Fig. 12). Like DDSC, Ca^2+^ increases also occurred before stimulation, but were significantly less frequent (Fig. 4c).

To examine whether these delayed Ca^2+^ transients and the plateau potential correspond to CaMKII activity, we performed simultaneous Ca^2+^ and CaMKII imaging by filling 2dV-Camui-transfected CA1 neurons with a Ca^2+^ indicator, Cal-590 (50 μM), through a patch pipette (Fig. 4d). The increases in Ca^2+^ during the BTSP protocol, which were probably due to back-propagating action potentials, were substantially smaller in amplitude and did not show associated CaMKII events (Fig. 4e). However, after BTSP, we observed CaMKII activity corresponding to DDSC associated with Ca^2+^ increases and the plateau potential (Fig. 4d). The Ca^2+^-triggered average of CaMKII activity clearly showed that the onset of CaMKII activation was temporally aligned with Ca^2+^ increases (Fig. 4e,f). Both Ca^2+^ and CaMKII events were larger during delayed events than those during the BTSP protocol (Fig. 4g,h).

**Extended Data Fig. 13:**
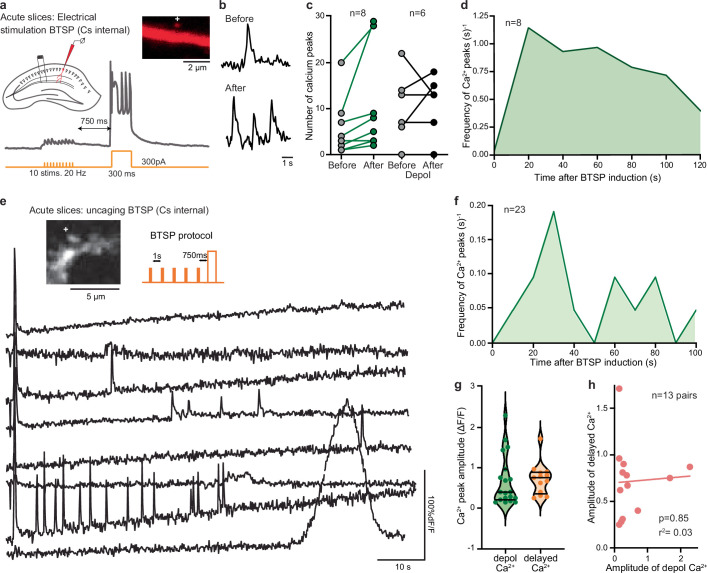
Similar delayed calcium events during BTSP in acute slices. **a**, Schematic showing electrical BTSP induction in acute hippocampal slices from adult mice. Bottom: representative BTSP protocol response. Right: representative dendrite filled with Cal-590. “+” depicts the BTSP stimulated spine. **b**, Representative Ca^2+^ activity imaged before and after electrical BTSP induction. **c**, Total number of Ca^2+^ peaks before and after electrical BTSP induction or depolarization only. **d**, The frequency of Ca^2+^ events as a function of the time after electrical BTSP protocol. The frequency peak appears around ~20–30 secs after BTSP protocol. **e**, Top: Representative dendritic image where BTSP was induced in one spine (+); Bottom: Representative dendritic Ca^2+^ traces after the uncaging BTSP protocol in acute slices. The traces show multiple delayed dendritic Ca^2+^ events after BTSP, in addition to a large event during the current injection of BTSP. **f**, Same as **(d)**, but after uncaging BTSP protocol. **g**, Ca^2+^ peak amplitude with Cs-internal is similar during depolarization and delayed activity. Data are presented as median and second quartile, along with individual values. Two-tailed unpaired t-test (t(30) = 0.22, p = 0.82). **h**, Correlation plot showing no correlation between Ca^2+^ activity during current injection and delayed calcium. (Two-sided Pearson R correlation, n = 13 pairs, r^2^ = 0.03, p = 0.85). The number of dendrites is mentioned in the figure panels.

Finally, we addressed whether delayed Ca^2+^ transients occur in acute slices prepared from adult mice (P45–P60). When we electrically induced BTSP using a published protocol^1^, we observed a large increase in the frequency of Ca^2+^ transients after BTSP induction (Extended Data Fig. 13a–d). However, depolarization alone did not induce such increases (Extended Data Fig. 13c). Similarly, we performed calcium imaging experiments with our single synapse uncaging BTSP protocol and found similar delayed calcium events following BTSP induction (Extended Data Fig. 13e–g). Thus, delayed Ca^2+^ transients can also be induced by BTSP in adult hippocampal slices.

## DDSC as an instructive signal for BTSP

**Fig. 5:**
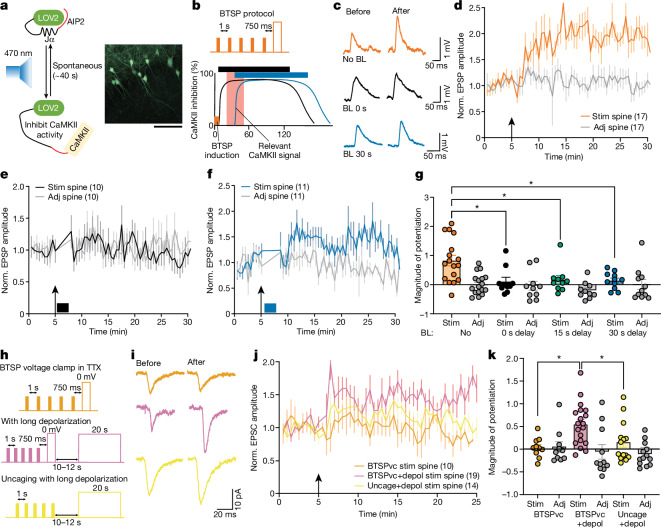
DDSC plays a crucial instructive role in BTSP. **a**, Left, schematic of the photoactivatable CaMKII inhibitor paAIP2, which inhibits CaMKII after blue light (BL, 470 nm) exposure and becomes inactive within about 40 s when BL is stopped. Right, paAIP2-P2A-mEGFP-labelled CA1 neurons. Scale bar, 50 μm. **b**, Schematic of two separate CaMKII inhibition experiments shown in **c**, **e** and **f**. CaMKII was inhibited for 2 mins, 0 s (black) or 30 s (blue) after the BTSP protocol (orange). **c**, Representative EPSP traces of a stimulated spine (average of ten recordings) before and after BTSP induction in paAIP2-labelled neurons for which no BL stimulation was given (orange), or BL was given with 0 s delay (black) or 30 s delay (blue). **d**–**g**, Normalized time course (**d**–**f**) and summary of magnitude of EPSP potentiation (25–30 min) (**g**) in stimulated and adjacent spines for no BL (*n* = 17; **d**), BL with 0 s delay (*n* = 10; **e**), BL with 15 s delay (*n* = 10) or BL with 30 s delay (*n* = 11; **f**). Two way ANOVA (*F*_7,68_ = 4.94, *P* = 0.0001) with Tukey’s multiple comparison test (all *P* values are shown in the source data file). **h**, Top, BTSP protocol in voltage clamp (BTSPvc), for which 5 uncaging pulses (1 Hz) were paired with depolarization to 0 mV for 300 ms with 750 ms delay in the presence of TTX. Middle, protocol to artificially induce delayed CaMKII activity by applying additional long (20 s) depolarization 10–12 s after BTSPvc (BTSPvc+depol). Bottom, 5 uncaging pulses were paired with long depolarization 10–12 s after uncaging (uncage+depol). **i**, Representative EPSC traces of stimulated spine (average of 10 recordings) before and 20 min after BTSPvc, BTSPvc+depol and uncage+depol. **j**,**k**, Normalized EPSC amplitude time course (**j**) and summary of magnitude of potentiation (**k**) of stimulated and adjacent spines during BTSPvc (*n* = 10), BTSPvc+depol (stim, *n* = 19; adj, *n* = 11) and uncage+depol (stim, *n* = 14; adj, *n* = 13). Two-way ANOVA (*F*_5,53_ = 4.9, *P* = 0.0009) with Tukey’s multiple comparison test (all *P* values are shown in the source data file). The data in **d**–**g**, **j** and **k** are presented as the mean ± s.e.m. Number of cells are mentioned in the panels in parentheses wherever appropriate. Arrows depict BTSP, BTSPvc or uncaging induction. *P < 0.05, ***P < 0.001.

**Extended Data Fig. 14:**
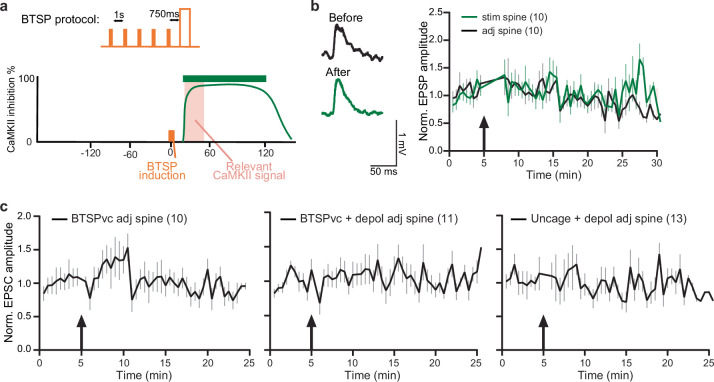
Additional optical CaMKII inhibition experiments during BTSP. **a**, Schematic of an additional CaMKII inhibition experiment shown in **(b)**. CaMKII was inhibited for 2 mins,15 s after the BTSP protocol (green). **b**, Left: Representative EPSP traces of a stimulated spine (average of 10 traces) before and after BTSP induction in paAIP2 labeled neurons. Right: Normalized EPSP time course in response to BTSP in stimulated and adjacent spines; Arrow depicts BTSP induction. **c**, Normalized EPSC time course in adjacent spines of BTSPvc, BTSPvc+depol and Uncage+depol experiments, showing no potentiation after BTSP induction. The number of cells is mentioned in the figure panel.

To test whether DDSC is essential in BTSP induction, we transduced neurons with the photoinducible competitive CaMKII inhibitor paAIP2 (ref. ^12^) using AAV, and inhibited CaMKII 0, 15 or 30 s after BTSP induction by illuminating with blue light (470 nm) (Fig. 5a,b and Extended Data Fig. 14a). Control CA1 neurons, which express paAIP2 but were not exposed to blue light, showed potentiation in EPSPs after the BTSP protocol (89 ± 20%, *n* = 17; Fig. 5c,d). However, when we inhibited CaMKII 0, 15 or 30 s after the BTSP protocol by blue light, significantly less synaptic potentiation was induced (11 ± 14% for blue light with 0 s delay, *n* = 10; −1.5 ± 8% for 15 s delay, *n* = 10; 14 ± 8% for 30 s delay, *n* = 11) (Fig. 5c,e–g and Extended Data Fig. 14b). Overall, these results show that CaMKII inhibition after inducing BTSP blocks synaptic potentiation and suggest that DDSC is necessary for BTSP induction.

We next examined whether DDSC is sufficient as an instructive signal in BTSP. We applied the BTSP protocol and inhibited plateau potentials through bath application of TTX (1 µM) in the voltage clamp and then artificially induced delayed Ca^2+^–CaMKII signalling through a long, delayed depolarization pulse (10–12 s delay, 20 s width)^8^ (Fig. 5h). In control neurons, a protocol similar to BTSP, a train of 5 uncaging pulses at 1 Hz paired with depolarization to 0 mV after a 750 ms delay, failed to induce synaptic potentiation (7 ± 8%, *n* = 10; Fig. 5i–k). However, in separate experiments, when the long depolarization pulse was delivered 10–12 s after the BTSP protocol, we observed a potentiation of EPSC amplitude in the stimulated spines (56.3 ± 16%, *n* = 19; Fig. 5i–k and Extended Data Fig. 14c), which suggested that depolarization-induced CaMKII^8^ provides instructive signals. When only uncaging pulses (without the depolarization during BTSP) were paired with a long-delayed depolarization pulse, we observed no significant change in EPSC amplitude in the stimulated spines (15.7 ± 11.4%, *n* = 14), which suggested that initial depolarization during BTSP also contributes to BTSP induction (Fig. 5h–k and Extended Data Fig. 14c). Taken together with results from the optogenetic experiments, these data indicate that delayed depolarization activates dendritic CaMKII, which provides an instructive signal essential for inducing synapse-specific BTSP.

## Intracellular calcium release underlies DDSC

**Fig. 6:**
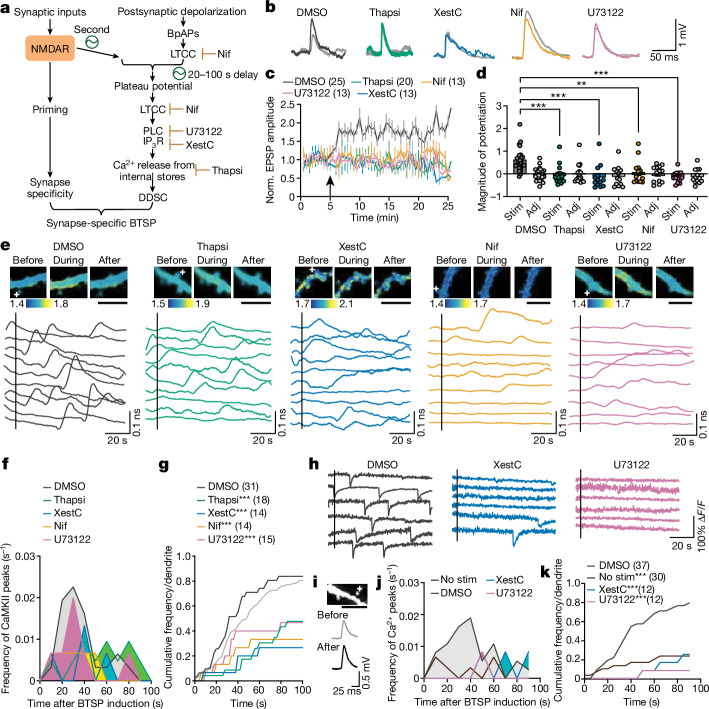
Calcium release from internal stores is required for BTSP and DDSC. **a**, A revised model of BTSP induction. Synaptic inputs activate NMDAR-dependent signalling to prime the stimulated synapses. Combined synaptic inputs and postsynaptic activation of LTCCs lead to delayed plateau potentials. Intracellular Ca^2+^ release is further facilitated by back-propagating action potentials (BpAPs) and activation of LTCCs. IP_3_-dependent calcium release from internal stores leads to delayed CaMKII activation (DDSC). DDSC acts as an instructive signal with an extended time window of 20–40 s. PLC, phospholipase C. **b**,**c**, Raw EPSP traces (**b**) (average of ten recordings) before and after BTSP induction and time course of synaptic potentiation (**c**) in stimulated spines in the presence of nifedipine (Nif), thapsigargin (Thapsi), XestC, U73122 or vehicle (DMSO). **d**, Summary of magnitude of EPSP potentiation for data in **c** (DMSO stim, *n* = 25, adj, *n* = 24; Thapsi stim, *n* = 20, adj, *n* = 18; Nif, *n* = 13; U73122, *n* = 13; XestC stim and adj, *n* = 13). The data are presented as the mean ± s.e.m. Two-way ANOVA (*F*_9,132_ = 6.419, *P* = 1.56 × 10^–7^) with Dunnett’s multiple comparison test (all *P* values are shown in the source data file). **e**, Top, fluorescence lifetime images of a dendrite before, during and after peak CaMKII activity in DMSO, Thapsi, XestC, Nif and U73122. Coloured bar, lifetime (ns). Bottom, representative smoothened dendritic lifetime traces showing DDSC under each condition. Scale bars, 2.5 μm (DMSO, XestC) or 5 μm (Thapsi, Nif, U73122). **f**, Time course of DDSC onset frequency under each condition. **g**, Cumulative histogram of **f**. Two-sided Kolmogorov–Smirnov test with Bonferroni’s correction for DMSO versus Thapsi, *P* = 7.4 × 10^–11^; DMSO versus XestC, *P* = 2.2 × 10^–13^; DMSO versus Nif, *P* = 4.3 × 10^–12^; and DMSO versus U73122, *P* = 7.4 × 10^–11^. BTSP data from Fig. 3 are also shown (light grey). **h**, Representative traces of ER-GCaMP6-210 experiments in cells treated with DMSO, XestC or U73122. **i**, Representative dendrite of a cell expressing the ER-GCaMP6-210 sensor with EPSP traces (bottom) showing successful BTSP induction. Scale bar, 5 μm. **j**, Time course of frequency of Ca^2+^ release events under no stim (brown), stim (DMSO), XestC and U73122 conditions. **k**, Cumulative histogram of **j**. Two-sided Kolmogorov–Smirnov test with Bonferroni’s correction for DMSO versus no stim, *P* = 0.0003; DMSO versus XestC, *P* = 0.0003; DMSO versus U73122, *P* = 0.000003. Number of cells or dendrites are mentioned in the panels in parentheses wherever appropriate. Plus symbol in images depict the BTSP-stimulated spine. **P < 0.01, ***P < 0.001.

A previous study showed that intracellular Ca^2+^ release from internal stores is required for in vivo BTSP-induced place cell formation^17^. Furthermore, intracellular store-induced Ca^2+^ release can be induced through IP_3_-dependent mechanisms^18^. L-type calcium channels (LTCCs), which are required for BTSP^1^, also play a pivotal part in Ca^2+^ release from internal stores in dendrites^19^. Thus, we investigated the role of LTCCs and intracellular Ca^2+^ release in BTSP and DDSC using various inhibitors. The following compounds were used: thapsigargin (1 μM), which depletes internal stores; xestospongin C (XestC, 1 μM), which inhibits the IP_3_ receptor (IP_3_R); U73122 (10 μM), which inhibits phospholipase C (the synthesis pathway of IP_3_ from PIP2); and nifedipine (10 μM), a blocker of LTCC. All these compounds significantly inhibited BTSP-induced synaptic potentiation compared to vehicle (DMSO) (Fig. 6a–d). Moreover, CaMKII imaging showed that DDSC was impaired in the presence of these drugs (Fig. 6e–g). Thus, LTCCs and IP_3_-dependent intracellular Ca^2+^ release is required for BTSP and DDSC.

**Extended Data Fig. 15:**
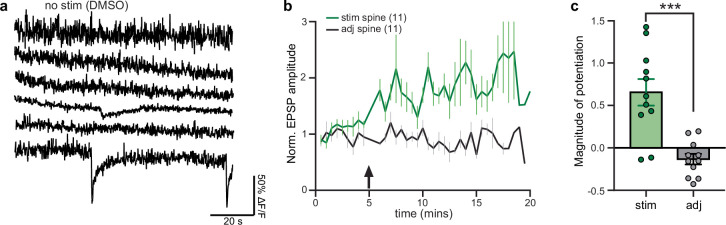
ER-GCaMP6-210 control imaging experiments and electrophysiology recordings. **a**, Representative traces of ER-GCaMP6-210 Ca^2+^ imaging in no-stim condition. **b**, Time course of synaptic potentiation following BTSP induction in the stimulated spines but not adjacent spines of ER-GCaMP6-210 labeled neurons. The arrow depicts BTSP induction. **c**, Summary of the magnitude of EPSP potentiation for data in **(b)** (n = 11). The data are presented as mean ± s.e.m. Two-tailed unpaired t-test (t(20) = 4.68, p = 0.0001). The number of cells is mentioned in the figure panel. ***p < 0.001.

Finally, to address whether DDSC is caused by Ca^2+^ release from the endoplasmic reticulum (ER), we directly imaged ER Ca^2+^ using an ER Ca^2+^ sensor (ER-GCaMP6-210)^20^. We observed transient decreases due to ER Ca^2+^ release. The release events increased after BTSP and were impaired by inhibiting the IP_3_ pathway through the use of XestC and U73122 (Fig. 6h–k and Extended Data Fig. 15). Overall, these experiments demonstrate that Ca^2+^ release from the ER has a crucial role in BTSP induction and DDSC.

## Discussion

BTSP has been a leading model for the induction of CA1 place cells. Our uncaging-evoked BTSP induced potentiation only in stimulated spines but not surrounding spines, which demonstrated that like Hebbian plasticity^21,22^, BTSP is an input-specific potentiation mechanism. This result supports previous studies suggesting that input-specific dendritic plasticity underlies place cell formation at a specific location^23–25^. Although the synapse-specific role of CaMKII in synaptic potentiation has been speculated, we did not observe any detectable spine-specific CaMKII activation during the BTSP protocol, even with our improved CaMKII sensor. However, we cannot rule out the possibility of some CaMKII activation below our detection limit during BTSP. In particular, a small fraction of CaMKII bound to GluN2B may provide synapse specificity^26,27^. Instead, we observed a global, delayed and stochastic CaMKII activity that spreads throughout the dendrite and nearby spines (DDSC). Our photoinhibition and voltage-clamp experiments indicated that DDSC has an essential role in BTSP induction. Ca^2+^ influx from LTCCs seems to lead to additional amplification by Ca^2+^-induced Ca^2+^ release, which produced sufficiently high Ca^2+^ increases for DDSC and BTSP. As DDSC, but not BTSP, is induced in basal dendrites, DDSC may not be linked with BTSP or additional inhibitory signalling may exist downstream of DDSC in basal dendrites. This may be related to a previous report suggesting that basal and apical dendrites have different properties of ER–mitochondrial coupling, which is essential for BTSP during place cell formation^17^. As the IP_3_ pathway can be regulated by Gq protein-coupled receptors, DDSC may receive additional regulation by neuromodulators.

It is mechanistically intriguing how presynaptic and postsynaptic activities are integrated over several hundreds of milliseconds and still result in NMDAR-dependent and synapse-specific plasticity. Although the time constant of CaMKII activation during Hebbian plasticity matches with the eligibility trace of BTSP, our study indicated that CaMKII is not the eligibility trace because CaMKII activation during BTSP was neither specific to the stimulated synapse nor active during the behavioural time scale (about 1 s). However, the global and delayed nature of DDSC would be consistent with its role as an instructive signal, although it provides a time window much larger than the proposed instructive signal^28^. As DDSC requires both presynaptic and postsynaptic components, additional biochemical signalling must exist upstream of CaMKII signalling (Fig. 6a). Furthermore, as BTSP-induced synaptic potentiation is spine-specific, the protocol needs to activate synapse-specific signalling to prime the stimulated spine, potentially through NMDAR-dependent signalling^14,29^. Overall, there are at least two time scales in this model: one for integrating presynaptic and postsynaptic inputs over the behavioural time scale (around 1 s), and the other for associating the synapse-specific priming signal and the instructive DDSC (10–100 s) (Fig. 6a). The integration of signals during the behavioural time scale does not need to encode synapse specificity, as this integration can occur in the dendrite and lead to delayed plateau potentials and DDSC (Fig. 6a). Instead, signal association during the slow time scale seems to give rise to the synapse specificity of BTSP. In vivo recording suggests that place cells are formed rapidly after spontaneous or experimentally induced plateau potentials^1,6^. However, it is unclear how the synapses are potentiated when generating this plateau potential during place cell formation. Our results indicated that upstream intracellular signalling may have started tens of seconds ahead of time to prime the stimulated synapses and to facilitate the generation of plateau potentials, which in turn contribute to synaptic potentiation during place cell formation.

## Methods

### Animals

All experimental procedures were approved and carried out in accordance with the regulations of the Max Planck Florida Institute for Neuroscience Animal Care and Use Committee as per the guidelines by the US National Institutes of Health. C57/B6 mice were used. The mice were kept in 12-h light–dark cycle at 18–21 °C with 40–50% humidity. We also used *Camk2a*^*T286A*^ mice to test the requirement of CaMKII in BTSP experiments^30^.

### Plasmid constructs

We fused two monomeric dimVenus (Venus(A206K,Y145W)) and mouse eGFP (eGFP(A206K)) to rat CaMKIIα subunit (2dV-Camuiα) (Addgene, 220366)^8,31^. T286A (Addgene, 220367) and T305D/T306D (Addgene, 220368) 2dV-Camuiα mutants were constructed by restriction digestion and ligation. For sensor screening in HeLa cells, we cotransfected the Calmodulin-1 (CaM) plasmid (Addgene, 240202). To do the ER calcium imaging experiments, we used the ER-GCaMP6-210 plasmid (Addgene, 86919).

### Organotypic hippocampal slice cultures and transfection

Organotypic hippocampal slices were prepared from wild-type or transgenic P4–P8 C57/B6 mouse pups of both sexes as previously described^32^. In brief, the animal was anaesthetized with isoflurane, after which it was quickly decapitated and the brain removed. The hippocampi were dissected and cut into 350-µm thick coronal hippocampal slices using a McIlwain tissue chopper (Ted Pella) and plated on hydrophilic PTFE membranes (Millicell, Millipore) fed by culture medium containing MEM medium (Life Technologies), 20% horse serum, 1 mM l-glutamine, 1 mM CaCl_2_, 2 mM MgSO_4_, 12.9 mM d-glucose, 5.2 mM NaHCO_3_, 30 mM HEPES, 0.075% ascorbic acid and 1 µg ml^–1^ insulin. The slices were incubated at 37 °C in 5% CO_2_. After 7–12 days in culture, CA1 pyramidal neurons were transfected using biolistic gene transfer with 1.0 µm gold beads (8–12 mg) coated with 2dV-Camuiα (50 μg)^33^. For CaMKII experiments, owing to the size of the CaMKII sensor, plasmid transfection using biolistic gene gun was the most effective. Transfection was done days in vitro day 7–10, and experiments were performed 2–7 days after transfection. The age of neurons would correspond to acute slices from juvenile animals^34^.

### Acute slice preparation

Male C57/B6 mice (P25–P35 or P45–P60) were sedated by isoflurane inhalation and perfused intracardially with a chilled choline chloride solution. The brain was removed and placed in the same choline chloride solution composed of 124 mM choline chloride, 2.5 mM KCl, 26 mM NaHCO_3_, 4 mM MgCl_2_, 1.2 mM NaH2PO_4_, 10 mM glucose and 0.5 mM CaCl_2_, pH 7.4 equilibrated with 95% O_2_ and 5% CO_2_. Coronal hippocampal slices (300 μm) from both hemispheres were cut using a vibratome (V1200, Leica) and maintained in a submerged chamber in artificial cerebrospinal fluid (ACSF; 127 mM NaCl, 2.5 mM KCl, 4 mM CaCl_2_, 25 mM NaHCO_3_, 1.25 mM NaH_2_PO_4_ and 25 mM glucose) at 32 °C for 1 h and then at room temperature in oxygenated ACSF.

### Two-photon glutamate uncaging

Two-photon glutamate uncaging was performed during BTSP and structural LTP experiments in organotypic hippocampal cultures and in acute hippocampal slices as previously described^35,36^. Experiments were performed in a small recirculating volume (about 8 ml) of continuously oxygenated ACSF containing 4 mM 4-methoxy-7-nitroindolinyl-caged-l-glutamate (MNI-caged glutamate). A Ti:Sapphire laser was tuned at a wavelength of 720 nm to uncage MNI-caged glutamate in a small region about 0.5 μm from the spine. For structural plasticity experiments, 30 uncaging pulses of 0.5 Hz train were given. The power of the laser was set to 2.7 mW measured at the objective. These structural plasticity experiments were performed in Mg^2+^-free ACSF (127 mM NaCl, 2.5 mM KCl, 4 mM CaCl_2_, 25 mM NaHCO_3_, 1.25 mM NaH_2_PO_4_ and 25 mM glucose) containing 1 μM TTX and 4 mM MNI-caged l-glutamate aerated with 95% O_2_ and 5% CO_2_. The BTSP experiments were performed in 2 mM Ca^2+^ and 1 mM Mg^2+^. Experiments were performed at room temperature (24–26 °C).

### Electrophysiology

Whole-cell patch-clamp electrophysiology experiments were combined with glutamate uncaging to induce BTSP at individual dendritic spines^36^. The cells were first visualized in a bright field, or for the labelled cells, epifluorescence microscopy. The patch pipette (with a tip resistance of 2–5 MΩ) included the K^+^-based internal solution containing 145 mM K-gluconate, 14 mM phosphocreatine, 4 mM NaCl, 0.3 mM NaGTP, 4 mM MgATP, 3 mM l-ascorbic acid, 50–100 µM Alexa-594 and 10 mM HEPES (pH 7.4, 294 mOsm). In BTSP experiments, the EPSPs were measured under the current-clamp mode by a patch-clamp amplifier (MC-700B, Molecular Devices) and digitizer (National Instruments). After 2–5 min of dye loading, fluorescence from Alexa-594 was used to find dendritic spines in 2pFLIM. Uncaging-evoked EPSPs were induced on 1–2 spines on a dendrite by MNI-glutamate uncaging, about 0.5 µm away from the tip of the spine. The uncaging-evoked EPSP amplitude was 0.4–2 mV. Some BTSP experiments were performed in voltage-clamp configuration, for which the cells were held at −70 mV. The baseline glutamate uncaging-evoked EPSC amplitude was between 5 and 20 pA. These voltage-clamp experiments were performed with Cs^+^-based internal solution containing 130 mM Cs-methanosulfonate, 6 mM KCl, 10 mM HEPES, 4 mM NaCl, 0.3 mM NaGTP, 4 mM MgATP and 14 mM Tris-phosphocreatine (BTSP voltage-clamp protocol). Experiments were performed at room temperature (24–26 °C). In the CaMKII imaging experiments, similar to the above experiments, Alexa-594 dye (Thermo Scientific, 100 µM) was loaded as a structural marker. For experiments using APV, TTX, nifedipine, xestospongin C and MK-801, slices were incubated with the drugs for more than 30 min before BTSP experiments and were applied throughout the recording. Control experiments were done each day before the addition of the drug. For DMSO control experiments, a different ACSF solution was prepared each day. In experiments with thapsigargin and U73122, slices were incubated with the drugs for at least 60 min before the experiments. For QX314 experiments, we started the recordings 3–5 min after establishing the whole-cell patch clamp, and only the cells in which the BTSP protocol did not elicit any spiking during the current injection were considered for further analyses. All drugs were purchased from Tocris Biosciences unless specified otherwise. EPSPs were measured before and after the induction of BTSP. In all whole-cell recordings, the series resistance was monitored to be between 10 and 40 MΩ throughout the recording.

### HeLa and HEK293FT cell maintenance, transfection and imaging

HeLa cells (American Type Culture Collection, CCL-2) and HEK293FT cells (Thermo Fisher) were grown in Dulbecco’s modified Eagle medium supplemented with 10% FBS at 37 °C in 5% CO_2_. Each CaMKIIα sensor plasmid was transfected into HeLa cells together with the Calmodulin-1 (CaM) plasmid (Addgene, 240202) at a 10:1 ratio using Lipofectamine 3000 (Invitrogen). Imaging was performed 24–48 h following transfection in a HEPES-buffered ACSF solution (20 mM HEPES pH 7.3, 130 mM NaCl, 2 mM NaHCO_3_, 25 mM d-glucose, 2.5 mM KCl and 1.25 mM NaH_2_PO_4_) with 2 mM CaCl_2_ and 2 mM MgCl_2_ by 2pFLIM as described below. When indicated, cells were stimulated with bath application of ionomycin (Tocris Biosciences) and then EGTA.

### Fluorescence-coupled size-exclusion chromatography

Expression vector DNA (2 µg) including Camuiα were transfected into HEK293S GnTI- cells (2 × 10^6^ cells per well in 6-well plates) cultured in FreeStyle 293 (Thermo Fisher) using TransIT2020 transfection reagent (Mirus Bio). Cells were collected 48 h after transfection, washed with ice-cold PBS and sonicated in 250 µl TBS (20 mM Tris-HCl (pH 8.0) and 200 mM NaCl) using a Misonix Sonicator 3000 (3 times, 30 s, power level of 9.0). The lysate was ultracentrifuged at 70,000 r.p.m. for 10 min (TLA110 rotor). The supernatant (20 µl) was loaded onto a Superose-6 size-exclusion chromatography column (10/300 GL; GE Healthcare), pre-equilibrated with TBS, and run at a flow rate of 0.4 ml min^–1^. The eluent from the Superose-6 column was detected using a fluorometer (RF-10AXL, Shimadzu) with the following settings: excitation, 475 nm; emission, 507 nm; time increment, 0.5 s; integration time, 1 s; and recording time, 75 min. The fluorescence-coupled size-exclusion chromatography data points were plotted using OriginPro graphic software (OriginLab v.9.5).

### Fluorescence correlation spectroscopy

HEK293FT cells (Thermo Fisher) were transfected with the plasmids using Lipofectamine 3000 (Thermo Fisher) and cultured for 2 days at 37 °C and 5% CO_2_. After washing the plate wells once in PBS buffer, the cells were lysed for 5 min with M-PER mammalian protein extraction reagent (Thermo Scientific), including Halt protease inhibitor (Thermo Scientific) and 5 mM EDTA. The lysates were centrifuged at 20,000*g* for 10 min and the supernatants were used for fluorescence correlation spectroscopy (FCS) measurement by diluting 2–15-fold in PBS buffer including the protease inhibitor. The FCS measurements were performed at 23 °C under a two-photon microscope without laser scanning, equipped with a Ti:Sapphire laser (Chameleon Ultra II, Coherent) tuned to a wavelength of 920 nm. The time-correlated single-photon counting data were collected for 60–120 s using a water-immersion objective (LUMPlanFL N ×60 NA 1.0 W, Olympus) directly immersed in 300 µl of the lysate solution, a single-photon counting board (Time Harp 260, PicoQuant) and a software of TTTR mode real-time correlator in TimeHarp 260 (v.3.0). Data analysis was performed using FoCuS-point software^37^.

### Optical CaMKII inhibition experiments

The CaMKII inhibition experiments were performed in organotypic hippocampal slices using previously described paAIP2 (ref. ^12^). In these experiments, slices were virally infected with 0.5–1 µl AAV mixture per slice (containing AAV9-Camk2a-Cre at 2 × 10^12^ vg per ml (1:1,000 dilution, Addgene (105558-AAV9) and rAAV8-DIO-CBA-paAIP2-mEGFP at 4.2 × 10^12^ vg per ml, UNC GTC Vector) at days in vitro 4–6 and imaged or patched at days in vitro 10–13. Cells with strong eGFP expression were used for experiments. Labelled cells were patched with the K^+^-based internal solution (see above) plus Alexa-594 dye in the patch pipette as described above. LED light stimulation (470 nm, M470L5, Thorlabs) was used to activate paAIP2.

### Two-photon microscopy and 2pFLIM

Custom-built two-photon fluorescence lifetime imaging microscopy was used to perform 2pFLIM as previously described^38^. 2pFLIM imaging was performed using a Ti:Sapphire laser (Coherent, Chameleon or Spark Alcor 920 nm (Spark Lasers)) at a wavelength of 920 nm with a power of 1.0–1.4 mW. Fluorescence emission was collected using a water-immersion objective (×60, NA 0.9, Olympus), divided with a dichroic mirror (565 nm) and detected with two separated photoelectron multiplier tubes placed after the wavelength filters (Chroma, 510/70-2p for green and 620/90-2p for red). Both red and green fluorescence was detected with photoelectron multiplier tubes with a low transfer time spread (H7422P40; Hamamatsu). Photon counting for fluorescence lifetime imaging was performed using a time-correlated single-photon counting board (Time-harp 260, Pico-Quant) using custom software (https://github.com/ryoheiyasuda/FLIMage_public). 2pFLIM images were collected at 64 × 64 pixels at the frame rate of 7.8 Hz (128 ms per frame), and the time course was filtered with a moving average over 30 frames. A second Ti:Sapphire laser tuned at a wavelength of 720 nm was used to uncage MNI-caged glutamate.

### Ca^2+^ imaging

Ca^2+^ imaging was performed by loading calcium dyes Cal-590 (50–100 μM, AAT Bioquest) together with a structural marker Alexa-488 (100 μM, Thermo Fisher Scientific). The Ca^2+^ sensor intensity measurements were collected at 64 × 64 pixels at the frame rate of 7.8 Hz with 2pFLIM (lifetime information was not used). The Ca^2+^ response was calculated by normalizing the intensity with the intensity of Alexa-488. The membrane voltage was also recorded during Ca^2+^ imaging under the current-clamp mode. In a subset of experiments, uncaging-evoked EPSPs were measured before and after BTSP induction. For simultaneous Ca^2+^ and CaMKII imaging experiments, Ca^2+^ was normalized to the average of the first 100 frames before the induction of BTSP. For Ca^2+^ imaging experiments in acute hippocampal slices, we performed a whole-cell patch clamp with electrodes (4–6 MΩ) loaded with Cs^+^-based internal solution (see above) plus Cal-590 (50 µM). We measured the baseline Ca^2+^ for 2–4 min and then applied a published BTSP protocol^1^, whereby Schaffer collaterals were stimulated with bipolar electrodes 10 times at 20 Hz and paired with postsynaptic current injection (300 pA for 300 ms) with a delay of 750 ms for 5 times. Then, Ca^2+^ imaging was resumed for another 2–4 mins. The Ca^2+^ events were detected using a custom Python code, whereby 3 times the standard deviation of the baseline noise was used as a detection threshold after the subtraction of the basal trend line obtained by linear regression.

### 2pFLIM analysis

2pFLIM analysis was performed as previously described^39^. To measure the fraction of the donor that was undergoing FRET with the acceptor (binding fraction), we fit a fluorescence lifetime curve summing all pixels over an entire image with a double exponential function convolved with the Gaussian pulse response function as follows:

1 $$F(t)={F}_{0}[{P}_{{\rm{D}}}\,H(t,{t}_{0},{\tau }_{{\rm{D}}},{\tau }_{{\rm{G}}})+{P}_{{\rm{AD}}}\,H(t,{t}_{0},{\tau }_{{\rm{AD}}},{\tau }_{{\rm{G}}})]$$

where *τ*_AD_ is the fluorescence lifetime of the donor bound with the acceptor, *P*_D_ and *P*_AD_ are the fraction of free donor and donor undergoing FRET with the acceptor, respectively, and *H*(*t*) is a fluorescence lifetime curve with a single exponential function convolved with the Gaussian pulse response function:

2 $$H(t,\,{t}_{0},\,{t}_{{\rm{D}}},\,{t}_{{\rm{G}}})=\frac{1}{2}\exp \left(\frac{{\tau }_{{\rm{G}}}^{2}}{2{\tau }_{{\rm{D}}}^{2}}-\frac{t-{t}_{0}}{{\tau }_{D}}\right){\rm{e}}{\rm{r}}{\rm{f}}{\rm{c}}\left(\frac{{\tau }_{{\rm{G}}}^{2}-{\tau }_{{\rm{D}}}(t-{t}_{0})}{\surd 2{\tau }_{{\rm{D}}}{\tau }_{{\rm{G}}}}\right)$$

in which *τ*_D_ is the fluorescence lifetime of the free donor, *τ*_G_ is the width of the Gaussian pulse response function, *F*_0_ is the peak fluorescence before convolution and *t*_0_ is the time offset, and erfc is the complementary error function.

To generate the fluorescence lifetime image, we calculated the mean photon arrival time, <*t*>, in each pixel as follows:

$$ < t > \,=\int tF(t){\rm{d}}t/\int F(t){\rm{d}}t,$$

Then, the mean photon arrival time was related to the mean fluorescence lifetime, <*τ*>, by an offset arrival time, *t*_0_, which was obtained by fitting the entire image as follows:

$$ < \tau  > = < t > -{t}_{0}.$$

For analysing fluorescence lifetime in regions of interests (ROIs) (spines or dendrites), we calculated the fluorescence lifetime by fitting the decay curve with equation (1), assuming *τ*_D_, *τ*_AD_, *τ*_G_ and *t*_0_ are constants within each image session. To measure the CaMKII time of occurrence and peak lifetime change in BTSP and control experiments, the raw traces were first normalized using the first 100 frames as baseline and then the normalized data were smoothened using a moving average of 60 data points. Following this processing, the time of CaMKII peak and amplitude was manually calculated on individual CaMKII traces.

### Statistics and reproducibility

All values are presented as the mean ± s.e.m. unless otherwise noted. The number of independent measurements or cells (*n*) is indicated in figures or figure legends. For electrophysiology experiments, the recordings were performed on 9–26 neurons (1 neuron per slice) from at least 2 different litters. For imaging experiments, the experiments were independently performed on 12–82 dendrites from at least 5 neurons (1 neuron per slice) from at least 2 different litters. For pharmacology experiments, 1–2 control experiments were performed on the same slices before the specific drug was added to the ACSF. In experiments for which DMSO was used as a vehicle, we performed control experiments on different days but on the slices made from the same batch as used in the experiments. Unpaired two-tailed Student’s *t*-test was used to compare two independent samples. Paired two-tailed Student’s *t*-test was used to compare dependent variables (before–after, soma–dendrite). One-way ANOVA followed by Dunnett’s multiple comparison test was used to compare more than two independent samples. Two-way ANOVA followed by Dunnett’s or Tukey’s multiple comparison test was used to compare grouped datasets. Correlation analysis was done by computing Pearson correlation coefficients. Data were organized in Microsoft Excel (v.2016). Data smoothening, statistical tests and *P* values are noted in each figure legend and were computed using GraphPad Prism (v.7.03, 9.5). Schematics of Figs. 1a, 3a and 5a and Extended Data Fig. 13 were created using Microsoft PowerPoint (v.2016) and Adobe Illustrator (v.27.9.1).

### Reporting summary

Further information on research design is available in the Nature Portfolio Reporting Summary linked to this article.

## Online content

Any methods, additional references, Nature Portfolio reporting summaries, source data, extended data, supplementary information, acknowledgements, peer review information; details of author contributions and competing interests; and statements of data and code availability are available at 10.1038/s41586-024-08021-8.

## Supplementary information

Supplementary Note Characterization of new CaMKII sensors.

Reporting Summary

## Data Availability

Source data associated with the figures presented in this paper are available at Synapse (https://www.synapse.org/Synapse:syn61441319/wiki/628796).

## Code availability

The FLIMage software for controlling FLIM hardware and analysing FLIM data is available at GitHub (https://github.com/ryoheiyasuda/flimage_public.git).
