# Author Correction: Dendritic, delayed, stochastic CaMKII activation in behavioural time scale plasticity

**DOI:** 10.1038/s41586-025-09295-2

**Published:** 2025-07-30

**Authors:** Anant Jain, Yoshihisa Nakahata, Tristano Pancani, Tetsuya Watabe, Polina Rusina, Kelly South, Kengo Adachi, Long Yan, Noriko Simorowski, Hiro Furukawa, Ryohei Yasuda

**Affiliations:** 1 Neuronal Signal Transduction Group, Max Planck Florida Institute for Neuroscience, Jupiter, FL USA; 2 W.M. Keck Structural Biology Laboratory, Cold Spring Harbor Laboratory, Cold Spring Harbor, NY USA; 3 Present Address: Centre for High Impact Neuroscience and Translational Applications (CHINTA), TCG CREST, Kolkata, India

**Keywords:** Cellular neuroscience, Long-term potentiation

Correction to: *Nature* 10.1038/s41586-024-08021-8 Published 9 October 2024

In the version of the article initially published, in the “Plasmid constructs” section of the Methods, the sentence “For sensor screening in HeLa cells, we cotransfected the Calmodulin-1 (CaM) plasmid (Addgene, 240202)” has been added before the sentence “To do the ER calcium imaging experiments, we used the ER-GCaMP6-210 plasmid (Addgene, 86919)”. In the section “HeLa and HEK293FT cell maintenance, transfection and imaging” of the Methods, the sentence “Plasmids were transfected into HeLa cells using Lipofectamine 3000 (Invitrogen)” has been replaced with “Each CaMKIIα sensor plasmid was transfected into HeLa cells together with the Calmodulin-1 (CaM) plasmid (Addgene, 240202) at a 10:1 ratio using Lipofectamine 3000 (Invitrogen)”. These corrections have been made to the HTML and PDF versions of the article and do not affect any results or conclusions.

