# Supplementary material for: Dendritic, delayed, stochastic CaMKII activation in behavioural time scale plasticity
Source: Nature. 2024 Oct 9;635(8037):151–9. doi: 10.1038/s41586-024-08021-8 (PMC11540904; doi:10.1038/s41586-024-08021-8)
Supplement: Supplementary file 1 — Characterization of new CaMKII sensors. [file 41586_2024_8021_MOESM1_ESM.docx]

**Characterization of new CaMKII sensors**

**Sensor Screening in HeLa cells**

Original Green-Camuiα sensor (1dV-Camui) is made of CaMKIIα subunit fused with monomeric dimVenus (Venus_A206K, Y145W_ or dV) and mEGFP (EGFP_A206K_) at N- and C-termini, respectively. To improve the sensor, we screened several modified sensors (**Extended data 2**) ^8^. We tested Camui with multiple acceptors (2dV-Camui and 3dV-Camui). Also, we tested a variant in which dimVenus is replaced by ShadowY (ShY) ^12^, which has an even lower quantum yield compared to dimVenus (1ShY-Camui and 2ShY-Camui).

In HeLa cells, we measured fluorescence lifetime changes in response to the bath application of ionomycin (3 µM) using 2pFLIM. Then, the decay kinetics of the sensors was measured by applying EGTA (8 mM). We found that all sensors show qualitatively similar kinetics (**Extended data 2b, c**). For dimVenus, we found a larger number of accepters provide a better signal and lower basal fluorescence lifetime, due to high FRET efficiency (**Extended data 2d, e**). However, Camui with ShY did not show a clear correlation between the number of accepters and FRET signals (**Extended data** **2d, e**).

While 3dV-Camui provided the best signal among these sensors, we decided not to use this sensor because it did not express well in neurons. Among others, we found that 2dV-Camui showed the best signal-to-background ratio (**Extended data. 2b,c**), and thus further analyzed this sensor.

To validate 2dV-Camui sensor, we analyzed their mutants (**Extended data 3**). When we mutated an autophosphorylation site that prolongs CaMKII activation (T286A) ^10,16,40-42^, the sensor signal was substantially reduced (**Extended data 3**). Furthermore, when we eliminated calmodulin binding required for CaMKII activation (T305D and T306D) ^43,44^, the sensor signal was abolished (**Extended data 3**). These results are consistent with previously described CaMKII behavior ^16^.

**Fluctuation correlation analysis**

CaMKII holoenzyme consists of dodecamer. Thus, we next studied if they can form dodecamer using fluctuation correlation spectroscopy (FCS) of the lysate of HEK293FT cells expressing CaMKII sensors (**Extended data 4**). FCS allows us to measure the diffusion speed of fluorescence molecules by measuring the time correlation of the fluorescence in a focused laser spot. Since diffusion is sensitive to the hydrodynamic diameter, we can estimate the size of fluorescence particles in the lysate ^45^.

We found that all EGFP-CaMKII, 1dV-Camuiα, 2dV-Camui, 3dV-Camui, 1ShY-Camui, 2ShY-Camui showed similar diffusion constant (~10 µm^2^/s), suggesting that increasing the number of accepters do not affect the hydrodynamic diameter (**Extended data 4a**). In comparison, the diffusion constant of EGFP (84 ± 2.9 µm^2^/s) and monomeric CaMKIIα (truncated CaMKIIα[1-306]) (62 ± 2.0 µm^2^/s) was much higher consistent with their smaller hydrodynamic diameters ^45^.

The diffusion speed of the sensors is somewhat smaller than previously measured diffusion of non-labeled CaMKII by dynamic light scattering (25 µm^2^/s) ^46^, perhaps because of the fluorophore fusion. To further examine if they can co-polymerize with non-labeled CaMKII, we co-expressed non-labeled CaMKII together with CaMKII sensors with different ratios. We found that, as the ratio of non-labeled CaMKII increases, the diffusion constant becomes higher, suggesting that they can copolymerize (**Extended data 4a, b**). Also, the y-intercept, which denotes the diffusion of non-labeled CaMKII, shows the diffusion constant similar to the one previously measured ^46^.

Thus, overall, these experiments suggest that the fusion of multiple accepters do not affect the hydrodynamic radius of the molecule, suggesting that they can form normal size of holoenzyme. Also, the new CaMKII sensor (2dV-Camui) can copolymerize with non-labeled CaMKIIα.

**Fluorescence coupled size-exclusion chromatography assay**

Next, we performed fluorescence coupled size-exclusion chromatography (FSEC) to measure the approximate size of the holoenzyme. Before testing the new sensors, we examined Green Camuiα, and EGFP-tagged CaMKIIα. As reported before ^8,31^, Green Camuiα shows similar peak retention time with EGFP-tagged CaMKIIα, suggesting that Green Camuiα is a dodecamer ^31^ (**Extended data 5**). Notably, when we tested Green Camuiβ, which uses CaMKIIβ instead of CaMKIIα, we observed an additional peak corresponding to smaller complex (**Extended data 5).** When we ran 2dV-Camui on the column, we found that this sensor showed a single peak time similar to EGFP-CaMKIIα and 1dV-Camuiα. Thus, taken together with FCS analysis, we concluded that 2dV-Camuiα can form dodecamer holoenzyme.

**Characterization of 2dV-Camui with 2-photon glutamate uncaging**

Finally, we tested 2dV-Camui in dendritic spines of pyramidal CA1 neurons in organotypic hippocampal slices. We applied 30 pulses of 2-photon glutamate uncaging in the absence of extracellular Mg^2+^. Consistent with previous study ^10^, we found that 2dV-Camui is activated in a step-wise manner in response to each uncaging pulse in the stimulated spine, and then decayed with a fast time constant of 7.3 s. The decay time constant was similar to that of 1dV-Camui (7.9 s). These kinetics are consistent with our previous study using the original Green-Camuiα (1dV-Camui) ^10^. Furthermore, 2dV-Camui with T286A mutation showed much faster decay (0.74 s) (**Fig. 2**) and failed to integrate uncaging pulses (**Fig. 2**), again consistent with our previous work ^10^. Finally, we found that the sensor with T305D and T306D mutations abolished the response, as expected ^47^. Overall, our results indicate that 2dV-Camui provides signals with the kinetics similar to the original Green-Camuiα, but with ~2 fold higher sensitivity.

**References:**

40 Chao, L. H. *et al.* A mechanism for tunable autoinhibition in the structure of a human Ca2+/calmodulin- dependent kinase II holoenzyme. *Cell* **146**, 732-745 (2011). https://doi.org:10.1016/j.cell.2011.07.038

41 Hanson, P. I., Kapiloff, M. S., Lou, L. L., Rosenfeld, M. G. & Schulman, H. Expression of a multifunctional Ca2+/calmodulin-dependent protein kinase and mutational analysis of its autoregulation. *Neuron* **3**, 59-70 (1989). https://doi.org:10.1016/0896-6273(89)90115-3

42 Hanson, P. I., Meyer, T., Stryer, L. & Schulman, H. Dual role of calmodulin in autophosphorylation of multifunctional CaM kinase may underlie decoding of calcium signals. *Neuron* **12**, 943-956 (1994). https://doi.org:10.1016/0896-6273(94)90306-9

43 Lou, L. L. & Schulman, H. Distinct autophosphorylation sites sequentially produce autonomy and inhibition of the multifunctional Ca2+/calmodulin-dependent protein kinase. *J Neurosci* **9**, 2020-2032 (1989). https://doi.org:10.1523/JNEUROSCI.09-06-02020.1989

44 Jama, A. M., Fenton, J., Robertson, S. D. & Torok, K. Time-dependent autoinactivation of phospho-Thr286-alphaCa2+/calmodulin-dependent protein kinase II. *J Biol Chem* **284**, 28146-28155 (2009). https://doi.org:10.1074/jbc.M109.005900

45 Chen, Y., Muller, J. D., Ruan, Q. & Gratton, E. Molecular brightness characterization of EGFP in vivo by fluorescence fluctuation spectroscopy. *Biophys J* **82**, 133-144 (2002). https://doi.org:10.1016/S0006-3495(02)75380-0

46 Gaertner, T. R. *et al.* Comparative analyses of the three-dimensional structures and enzymatic properties of alpha, beta, gamma and delta isoforms of Ca2+-calmodulin-dependent protein kinase II. *J Biol Chem* **279**, 12484-12494 (2004). https://doi.org:10.1074/jbc.M313597200

47 Pi, H. J., Otmakhov, N., Lemelin, D., De Koninck, P. & Lisman, J. Autonomous CaMKII can promote either long-term potentiation or long-term depression, depending on the state of T305/T306 phosphorylation. *J Neurosci* **30**, 8704-8709 (2010). https://doi.org:10.1523/JNEUROSCI.0133-10.2010
