# Supplementary material for: Dendritic, delayed, stochastic CaMKII activation in behavioural time scale plasticity
Source: Nature. 2024 Oct 9;635(8037):151–9. doi: 10.1038/s41586-024-08021-8 (PMC11540904; doi:10.1038/s41586-024-08021-8)
Supplement: Supplementary file 2 — Reporting Summary [file 41586_2024_8021_MOESM2_ESM.pdf]

nature portfolio

Corresponding author(s): Ryohei Yasuda

Last updated by author(s): 07/01/2024

Reporting Summary

Nature Portfolio wishes to improve the reproducibility of the work that we publish. This form provides structure for consistency and transparency in reporting. For further information on Nature Portfolio policies, see our Editorial Policies and the Editorial Policy Checklist.

Statistics

For all statistical analyses, confirm that the following items are present in the figure legend, table legend, main text, or Methods section.

n/a	Confirmed	
☐	☒	The exact sample size (*n*) for each experimental group/condition, given as a discrete number and unit of measurement
☐	☒	A statement on whether measurements were taken from distinct samples or whether the same sample was measured repeatedly
☐	☒	The statistical test(s) used AND whether they are one- or two-sided<br>*Only common tests should be described solely by name; describe more complex techniques in the Methods section.*
☒	☐	A description of all covariates tested
☐	☒	A description of any assumptions or corrections, such as tests of normality and adjustment for multiple comparisons
☐	☒	A full description of the statistical parameters including central tendency (e.g. means) or other basic estimates (e.g. regression coefficient) AND variation (e.g. standard deviation) or associated estimates of uncertainty (e.g. confidence intervals)
☐	☒	For null hypothesis testing, the test statistic (e.g. F, t, r) with confidence intervals, effect sizes, degrees of freedom and P value noted<br>*Give P values as exact values whenever suitable.*
☒	☐	For Bayesian analysis, information on the choice of priors and Markov chain Monte Carlo settings
☒	☐	For hierarchical and complex designs, identification of the appropriate level for tests and full reporting of outcomes
☐	☒	Estimates of effect sizes (e.g. Cohen's *d*, Pearson's *r*), indicating how they were calculated

Our web collection on statistics for biologists contains articles on many of the points above.

Software and code

Policy information about availability of computer code

Data collection	The electrophysiology and imaging experiments were performed on a custom built two-photon fluorescence lifetime microscope. The details of individual components are mentioned in the methods section. Photon counting for fluorescence lifetime imaging was performed using a time-correlated single-photon counting board (Time-harp 260, Pico-Quant) using custom software developed in C# (https://github.com/ryoheiyasuda/FLIMage_public). Imaging and electrophysiology data collection was also done in FLIMage.
Data analysis	Imaging and electrophysiology data analysis was done in custom written FLIMage software. Microsoft excel and Graphpad Prism (v7.3 and v9.5) was used to perform statistical test and plotting of graphs. The fluorescence-coupled size-exclusion chromatography data was plotted using OriginPro graphic software (OriginLab v9.5)

For manuscripts utilizing custom algorithms or software that are central to the research but not yet described in published literature, software must be made available to editors and reviewers. We strongly encourage code deposition in a community repository (e.g. GitHub). See the Nature Portfolio guidelines for submitting code & software for further information.

Data

Policy information about availability of data

All manuscripts must include a data availability statement. This statement should provide the following information, where applicable:

- Accession codes, unique identifiers, or web links for publicly available datasets
- A description of any restrictions on data availability
- For clinical datasets or third party data, please ensure that the statement adheres to our policy

All source data associated with the data presented in this paper has been uploaded to Synapse.org. Additional data that support the findings of this study are available from the corresponding author RY (ryohei.yasuda@mpfi.org) upon request.

Research involving human participants, their data, or biological material

Policy information about studies with human participants or human data. See also policy information about sex, gender (identity/presentation), and sexual orientation and race, ethnicity and racism.

Reporting on sex and gender	NA
Reporting on race, ethnicity, or other socially relevant groupings	NA
Population characteristics	NA
Recruitment	NA
Ethics oversight	NA

Note that full information on the approval of the study protocol must also be provided in the manuscript.

Field-specific reporting

Please select the one below that is the best fit for your research. If you are not sure, read the appropriate sections before making your selection.

☒ Life sciences ☐ Behavioural & social sciences ☐ Ecological, evolutionary & environmental sciences

For a reference copy of the document with all sections, see nature.com/documents/nr-reporting-summary-flat.pdf

Life sciences study design

All studies must disclose on these points even when the disclosure is negative.

Sample size	The sufficiency of the sample size was determined by using power calculation based on the mean and standard deviation of the major end points in each experiment to provide 90% power (type 1 error set at 0.05) for effect sizes of 15%.
Data exclusions	Pre-exclusion criteria for electrophysiology data was following - 1) data were excluded if the EPSP recording was less than 5 mins after BTSP induction. 2) if the holding current and access resistance were not comparable as before BTSP induction. In stimulated spines in BTSP experiment, both responsive and non-responsive data were included in each experiment. For CaMKII characterization experiments, only spines that showed successful structural LTP were included in the analysis.
Replication	Each experiment data was reproduced in different neurons from atl east 2 animals. In electrophysiology experiments, the recordings were independently performed 9-28 times in at least 2 batches of slices. For imaging, the experiments were independently performed in at least 12-84 dendrites from at least 5 neurons.<br>In pharmacology, majority of the replication attempts were successful and even those that were not replicated have been reported in the summary section. In other experiments, all replication attempts were successful.
Randomization	Randomization was done in pharmacology uncaging BTSP experiments, where control versus drug induced experiments were performed intermittantly. Randomization was also done during optical CaMKII manipulation experiments where the time when the Blue light stimulation begins was changed between two recordings.
Blinding	The experimenters were not blinded while doing the data acquisition and data analysis as the main finding that showed delayed CaMKII during BTSP was done together with electrophysiological recording. To overcome this, we performed several controls and have reported it in the manuscript.

Reporting for specific materials, systems and methods

We require information from authors about some types of materials, experimental systems and methods used in many studies. Here, indicate whether each material, system or method listed is relevant to your study. If you are not sure if a list item applies to your research, read the appropriate section before selecting a response.

Materials & experimental systems

n/a	Involved in the study
☒	☐ Antibodies
☐	☒ Eukaryotic cell lines
☒	☐ Palaeontology and archaeology
☐	☒ Animals and other organisms
☒	☐ Clinical data
☒	☐ Dual use research of concern
☒	☐ Plants

Methods

n/a	Involved in the study
☒	☐ ChIP-seq
☒	☐ Flow cytometry
☒	☐ MRI-based neuroimaging

Eukaryotic cell lines

Policy information about cell lines and Sex and Gender in Research

Cell line source(s)	The HeLa and HEK293FT cells were obtained directly from ATCC and Thermo Fisher respectively.
Authentication	The cells were not authenticated after arrival. 2nd and 3rd passage cells were used for experiments
Mycoplasma contamination	We confirm that all cell lines were tested negative for mycoplasma contamination
Commonly misidentified lines (See ICLAC register)	No commonly misidentified cell lines were used in the study

Animals and other research organisms

Policy information about studies involving animals; ARRIVE guidelines recommended for reporting animal research, and Sex and Gender in Research

Laboratory animals	4-8 day old C57/BL6 mouse pups were used to prepare hippocampal organotypic slices. For acute slices 25-60 day old C57/BL6 male mice were used. Camk2aT286A mice of either sex were used to test the requirement of CaMKII in BTSP experiments (PMID: 9452388). The mice were kept in a 12 hour light dark cycle at 65-70 degree F and 40-50% humidity
Wild animals	No wild animals were used in the study.
Reporting on sex	We prepared organotypic slice culture from both sexes but it was difficult to determine sex during the P4-P8 stage. Thus sex specific analysis was not performed in our study.
Field-collected samples	No field collected samples were used in the study.
Ethics oversight	All experimental procedures were approved and carried out in accordance with the regulations of the Max Planck Florida Institute for Neuroscience Animal Care and Use Committee as per the guidelines by the US National Institutes of Health.

Note that full information on the approval of the study protocol must also be provided in the manuscript.
